# Reducing the fatal attraction of nocturnal insects using tailored and shielded road lights

**DOI:** 10.1038/s42003-024-06304-4

**Published:** 2024-05-31

**Authors:** Manuel Dietenberger, Andreas Jechow, Gregor Kalinkat, Sibylle Schroer, Birte Saathoff, Franz Hölker

**Affiliations:** 1 Leibniz Institute of Freshwater Ecology and Inland Fisheries (IGB), Müggelseedamm 310, 12587 Berlin, Germany; 2 Institute of Biology, Freie Universität Berlin, Königin-Luise-Straße 1-3, 14195 Berlin, Germany; 3 Chair of Nature Conservation and Landscape Ecology, Albert-Ludwigs-Universität Freiburg, Stefan-Meier-Str. 76, 79104 Freiburg, Germany; 4 Department of Engineering, Brandenburg University of Applied Sciences, Magdeburger Str. 50, 14770 Brandenburg an der Havel, Germany; 5 Institute of Energy and Automation Technology, Technische Universität Berlin, Marchstraße 23, 10587 Berlin, Germany

**Keywords:** Conservation biology, Community ecology

## Abstract

The attraction of insects to artificial light is a global environmental problem with far-reaching implications for ecosystems. Since light pollution is rarely integrated into conservation approaches, effective mitigation strategies towards environmentally friendly lighting that drastically reduce insect attraction are urgently needed. Here, we tested novel luminaires in two experiments (i) at a controlled experimental field site and (ii) on streets within three municipalities. The luminaires are individually tailored to only emit light onto the target area and to reduce spill light. In addition, a customized shielding renders the light source nearly invisible beyond the lit area. We show that these novel luminaires significantly reduce the attraction effect on flying insects compared to different conventional luminaires with the same illuminance on the ground. This underlines the huge potential of spatially optimized lighting to help to bend the curve of global insect decline without compromising human safety aspects. A customized light distribution should therefore be part of sustainable future lighting concepts, most relevant in the vicinity of protected areas.

## Abstract (web summary)

A customized and shielded street lighting design significantly reduces the lethal attraction of flying insects in various environments. The approach harmonizes human needs with minimizing the ecological impact of artificial light at night.

## Introduction

The declines in abundance, species richness and biomass of several insect groups over the last decades represent a dramatic global trend^1–3^. Beside land-use change, pesticide use, climate change and habitat fragmentation, artificial light at night (ALAN) has been considered to be a major driver^4–6^. This is especially relevant for nocturnal insects, which represent roughly half of all insect species^7^. Alternating light and dark periods on a daily or seasonal time scale are important for the synchronization of many physiological and behavioral processes^8,9^. In this context, ALAN can interfere with the temporal organization of many organisms and disturbs the complex diel interactions and processes that sustain ecosystems^10–12^. A well-known and fundamental negative effect of ALAN is its attraction to many insects^13–15^, that withdraws them from their native habitats. Since ALAN is growing in intensity and spatial extent^16,17^, mitigation strategies for sensitive environments, especially protected areas, are urgently needed. Recently, many outdoor light sources, including road lights, are shifting to light-emitting diode (LED) technology, due to improvements in energy costs, luminous efficacy and color rendering^18^. This shift to LED alters the spectral and spatial distribution of ALAN, what may affect the attraction of flying insects from nearby habitats. However, the results of studies investigating the effects of different types of road lights vary greatly^19^. This is due to a pronounced diversity in spectral sensitivities and behavioral responses, but also because the spatial distribution was often not investigated. Thus, uncertainty remains as to which approaches are best suited for reducing the ecological impacts of ALAN on flying insects^20^. In this study, we primarily investigated the impact of spatial confinement and secondly of reducing illuminance (“dimming”) of road lighting on the attraction of flying insects. This was done by monitoring individual luminaires with flight interception traps on nights between March and October. Our study comprises two experiments with a total of four sites. At each of the sites, novel LED luminaires were tailored to only emit light onto the target area. An additional shielding minimizes spill light at large emission angles directly at the exit point of the radiation. These tailored and shielded LED road lights are nearly invisible beyond the lit area.

**Fig. 1:**
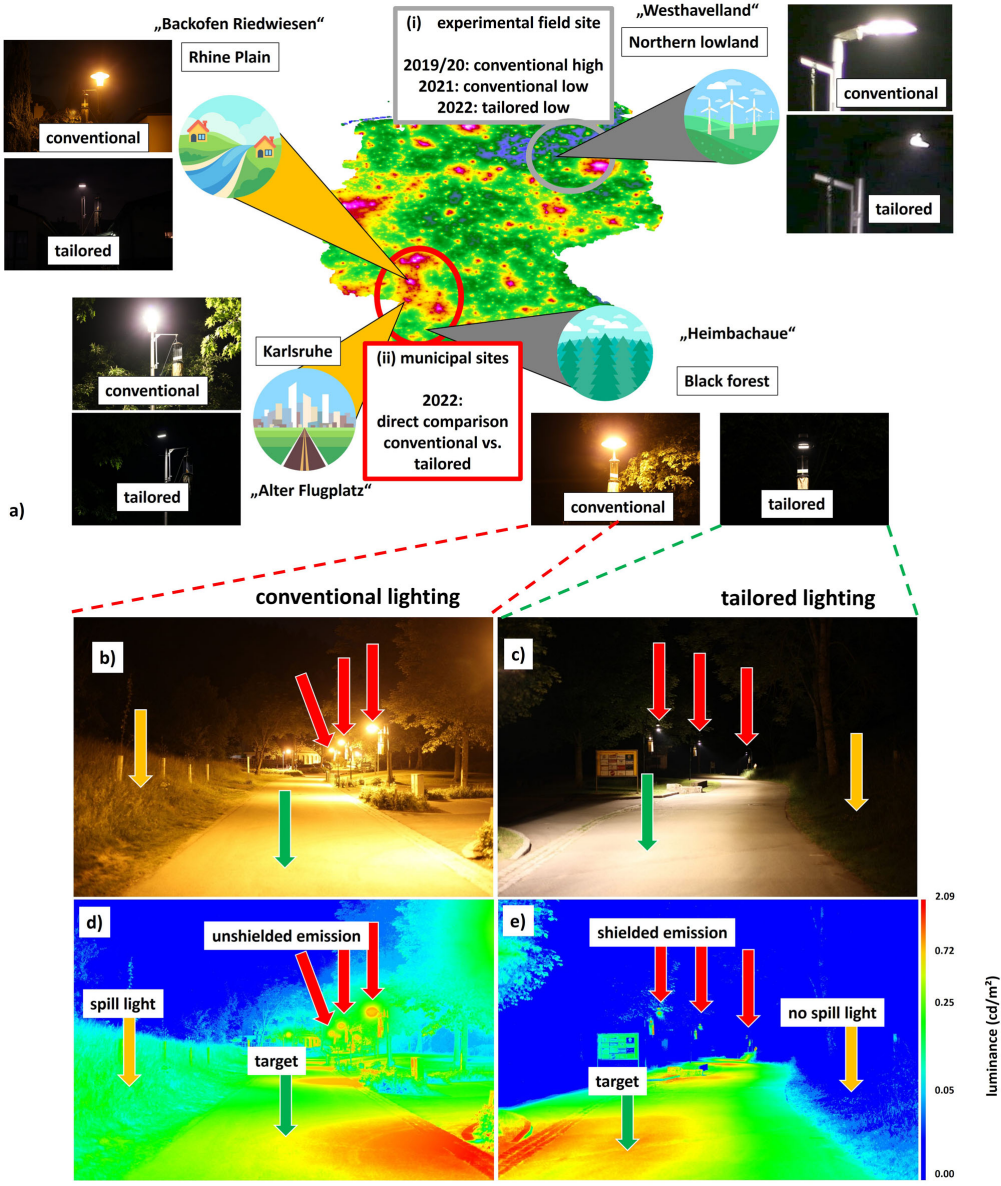
Experimental sites and Design. **a** Map of sampling sites, landscape context (iconized), skyglow (background map layer) and respective lighting treatment (insets) in Germany. The map depicts the four study sites on the outline of Germany using data from a skyglow model^51^; **1**. Experimental field site Westhavelland (upper right), rural site, very low skyglow (dark sky reserve), extensively managed grasslands, n = 12 luminaires per lit treatment, transition from conventional (LED, 3000 K) to tailored and shielded luminaires (LED, 3000 K)); plus dark control treatments (*n* = 12 unlit luminaires) **2**. Municipal site Alter Flugplatz Karlsruhe (lower left), urban site, sand and rough grassland, extreme skyglow, *n* = 5 luminaires per treatment, transition from conventional luminaires (LED, 4000 K) to tailored and shielded luminaires (LED, 4000 K) **3**. Municipal site Backofen Riedwiesen (upper left), suburban, very high skyglow, flood plain of Rhine river, *n* = 5 luminaires per treatment, transition from conventional (cylindrical HPS, 2000 K) to tailored and shielded luminaires (LED, 2700 K) **4**. Municipal site Heimbachaue (lower right), rural, black forest, low skyglow, *n* = 4 luminaires per treatment, transition from conventional (bell-shaped HPS, 2000 K) to tailored and shielded luminaires (LED, 2700 K). Effect of a tailored spatial light field and additional shielding at the municipal site Heimbachaue. Left images (**b**, **d**) show conventional luminaires and right images (**c**, **e**) show tailored and shielded luminaires. The upper row (**b**, **c**) shows RGB images, the lower row (**d**, **e**) calculated luminance maps from the images in the upper row. Images were obtained on the same night from the same position in the center of the test site. The tailored luminaires emit light only on the target area (green arrows), while reducing spill light into adjacent areas (yellow arrows). A further tailored shielding reduces the light directly at the luminaire itself (red arrows). While the conventional road lights are visible from large distances, producing the highest luminance at luminaire heads (**d**, red arrows), the tailored LEDs are nearly invisible from a distance (**e**, red arrows). See Supplementary Figs. 6–16 for further RGB/drone images and spectral measurements). Landscape icons were made by Freepik from www.flaticon.com.

(i) In the first multi-year experiment (2019–2022), we monitored individual road lights in three different light configurations (=treatments) with the same correlated color temperature (CCT 3000 K). The experiment took place at the controlled experimental field site “Westhavelland”. This field site comprises two separated sets of road lights in an isolated dark rural environment^21^. It has one set of twelve road lights that were lit as treatments, and one set of twelve road lights that were unlit as control treatments (Supplementary Figs. 1–4). In the first treatment (2019, 2020), we tested conventional LED luminaires at high illuminance (average illuminance on target area *E*_m_ = 30.6 lx). In the second treatment (2021), the illuminance of these conventional LED luminaires was reduced (“dimmed”) to 18% (*E*_m _= 5.6 lx). In the third treatment (2022), the conventional LED luminaires were replaced with the novel tailored and shielded LED luminaires at the same illuminance as the second treatment (*E*_m _= 5.2 lx, Fig. 1a). For spectral composition see Supplementary Fig. 5.

(ii) In the second experiment, the tailored and shielded lighting-approach was transferred to streets close to nature reserves at three municipal sites in Southern Germany (Fig. 1a). This was conducted simultaneously to the controlled experimental field site (i) in 2022. Here, we similarly installed the novel luminaires and compared them to the existing conventional lighting in terms of insect attraction using the same trapping technique. These novel luminaires represent an optimized solution for each site and were adapted to local site conditions (street width, height and spacing of luminaires), while keeping the illuminance at the road traffic compliant and nearly identical to the previously installed road lights. At one site, “Alter Flugplatz”, conventional LEDs were compared to tailored LEDs while keeping CCT constant at 4000 K (Supplementary Figs. 6–9), in line with the first experiment at the controlled experimental field site (i). At the two other sites, “Heimbachaue” and “Backofen Riedwiesen”, conventional high-pressure sodium (HPS) luminaires (CCT 2000K) were compared with tailored LED luminaires at a higher CCT (2700 K), reflecting an ongoing transition towards LEDs in our experimental design (Fig. 1b–e, Supplementary Figs. 10–16). The sites cover a gradient in skyglow (ALAN scattered back in the atmosphere), urbanization and lighting contexts and a range of different landscapes and ecosystems.

Together, these complimentary experimental set-ups allowed us to test three hypotheses, namely H1) that reducing illuminance (”dimming”) decreases attraction to a wide range of phototactic insect taxa at the experimental field site (i), H2) that a tailored spatial light field and shielding reduce insect attraction when keeping the illuminance on the target area constant regardless of the lighting situation and associated habitats at experimental (i) and municipal sites (ii) and H3) that the presence of ALAN and the applied modifications result in an altered community composition of attracted insects at experimental (i) and municipal sites (ii) by differently affecting insect taxa^22,23^. This additionally raises the question if there are any insect taxa especially relevant in a potential community shift.

## Results

### (i) Experimental field site Westhavelland

A total of 7240 Insects were caught over a period of four years at the controlled experimental field site in Westhavelland (Supplementary Table 1). This excludes one sampling event on 19.05. 2019, representing a mass emerge of *Nematocera* with more than 2000 individuals in some traps of the lit treatment in Westhavelland. In the lit treatments, most abundant insect groups caught over a 4-year period were *Nematocera* (3196), *Ephemeroptera* (754) and *Sternorrhyncha* (689). The total number of insects in the dark control treatments remained constant over the whole experiment (2019–2020: 430, 2021: 397, 2022: 421). At the unlit dark control treatments, *Coleoptera* (471) and *Nematocera* (332) were most abundant over four years. Comparing total insect numbers between light treatments and dark controls (Table 1, Supplementary Fig. 17), we observed a 7.7-fold higher attraction of insects with conventional LEDs at high illuminance in the first treatment (30.6 lx) than with the corresponding dark control treatment. Dimming of conventional LEDs to 5.6 lx in the second treatment lowered this factor slightly to 5.4 (Table 1). For the tailored and shielded luminaires in the third treatment at the lower illuminance (5.2 lx), insect attraction was reduced to only 1.3-fold compared to the associated dark control (Table 1). As these total numbers may be biased due to seasonal environmental differences between years, we performed linear mixed modeling.

**Table 1:** Total number of attracted insects at the experimental field site in Westhavelland

Treatment	1. Conventional High	1. Dark control	2. Conventional Low	2. Dark control	3. Tailored Low	3. Dark control
Total Number of Insects	3296	430	2130	397	566	421
Factor to dark control	7.7		5.4		1.3	

Treatment numbers refer to treatments of the same years. 1: Conventional High 2019-2020 (*n* = 14 samplings, LED 30.6 lx 3000 K). 2: Conventional Low 2021 (*n* = 15 samplings, LED 5.6 lx 3000 K). 3: Tailored Low 2022 (*n* = 16 samplings, LED 5.2 lx 3000 K). Dark controls were sampled simultaneously with the lit treatments. 12 luminaires per treatment.

#### a) Abundance

**Fig. 2:**
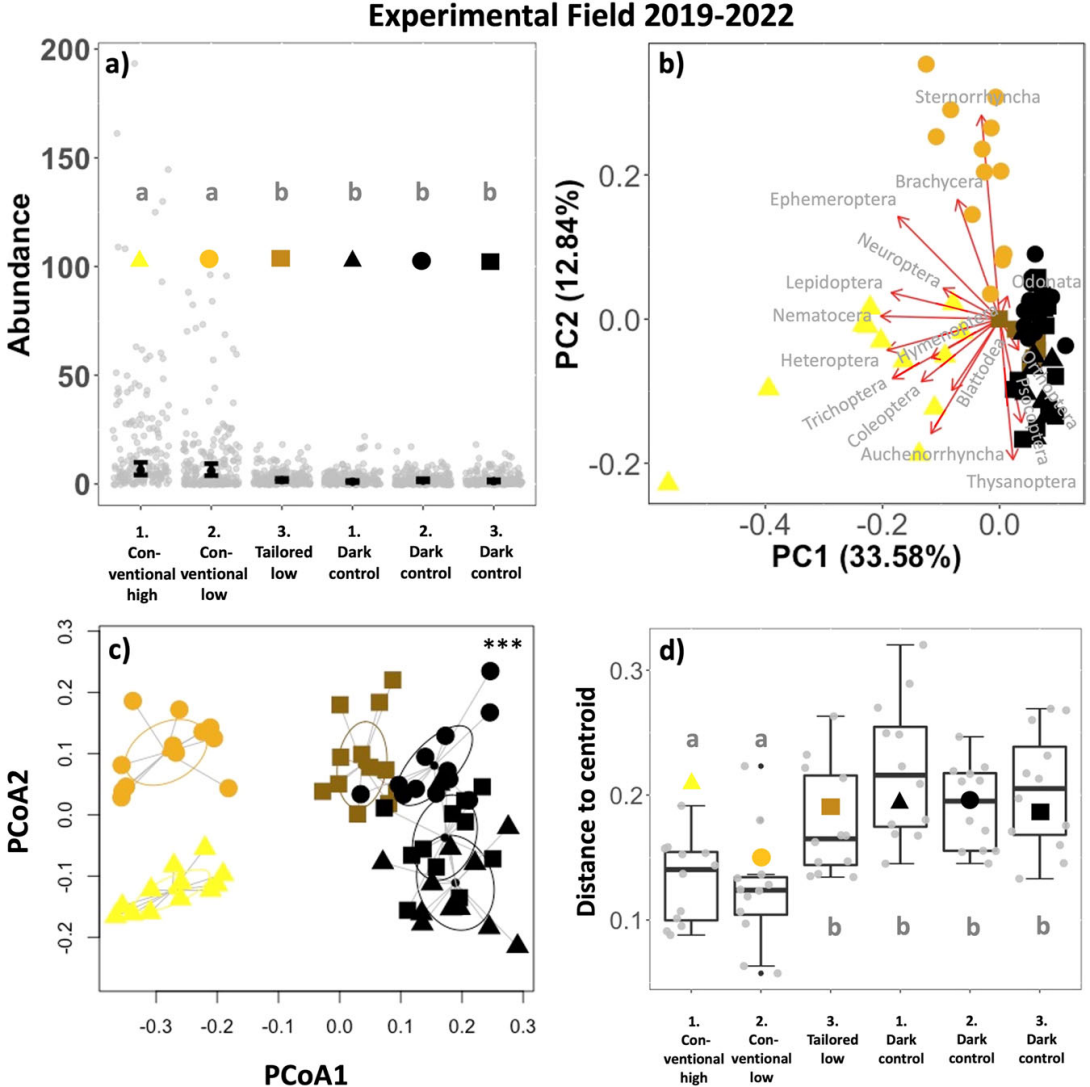
Experimental field site Westhavelland. **a** Total number of individuals per road light and night (Abundance, gray points) in different treatments (symbols, *n* = 12 luminaires per treatment). Predictions (black points) and 95% confidence intervals (error bars) based on a generalized linear regression model. Different letters indicate significant differences, similar letters mean no significant differences (Tukey post hoc test). Treatment numbers refer to treatments of the same years. 1: Conventional High 2019–2020 (*n* = 14 samplings, LED 30.6 lx 3000 K). 2: Conventional Low 2021 (*n* = 15 samplings, LED 5.6 lx 3000 K). 3: Tailored Low 2022 (*n* = 16 samplings, LED 5.2 lx 3000 K). Dark controls were sampled simultaneously to the lit treatments. **b** PCA Principal component analysis of multiple luminaires (*n* = 12) grouped by treatment (symbols). Arrow length represents loadings of individual insect taxa. **c** PERMANOVA Permutational Multivariate Analysis of Variances (df = 5, *R*^***2***^ = 0.633, *F* = 22.764, *p* = 0.001**) of multiple luminaires (*n* = 12) grouped by treatment (symbols). Ellipses based on standard deviation (SD). **d** Within group dispersion (community dispersion) of luminaires (*n* = 12) in the same treatments. Distances to centroids (gray points). Box plots show median (thick line), interquartile range (IQR) (box), outliers (black points), 1.5 × IQR (whiskers). Different letters indicate significant differences, similar letters mean no significant difference (Tukey post hoc test).

Linear regression of the number of insects attracted per light and night (abundance) showed that conventional LED luminaires in treatments 1 and 2 at two different illumination levels (high 30.6 lx and low 5.6 lx) attracted significantly more insects than the tailored and shielded luminaires at the low illumination level (5.2 lx) in treatment 3 and dark control treatments at the experimental field site in Westhavelland (Fig. 2a, Table 2). Dimming of conventional luminaires (treatment 2) did not have a significant effect (*p* = 0.812) but using luminaires with a spatially tailored light distribution and additional shielding (treatment 3) significantly reduced insect abundance (*p* < 0.001***). The nightly mean temperature had a significant positive effect on insect abundances, predicting higher insect abundance at higher temperatures across treatments (*p* < 0.001***). While the mean wind speed did negatively affect insect abundance in the flight interception traps (*p* < 0.001***), mean precipitation had no significant effect (*p* = 0.771). A pairwise comparison further revealed that tailored and shielded luminaires were not significantly different from any dark control treatment in terms of insect abundance (Supplementary Table 2).

**Table 2:** Effects of light treatments on insect abundance at the experimental field site Westhavelland (generalized linear model)

Abundance ~	Estimate	SE	*Z*	*P*
1. Conventional High	0.35542	0.43186	0.823	0.410517
2. Conventional Low	−0.07195	0.31584	−0.228	0.819785
3. Tailored Low	−1.32383	0.28964	−4.571	4.86e-06***
1. Dark control	−1.83383	0.12246	−14.975	<2e-16***
2. Dark control	−1.43478	0.33045	−4.342	1.41e-05***
3. Dark control	−1.60846	0.30206	−5.325	1.01e-07***
Temperature (°C)	0.18957	0.02519	7.527	5.21e-14***
Wind (m/s)	−0.48714	0.13718	−3.551	0.000384***
Precipitation (mm)	−0.09418	0.32339	−0.291	0.770879

Treatment numbers refer to treatments of the same years. 1: Conventional High 2019-2020 (*n* = 14 samplings, LED 30.6 lx 3000 K). 2: Conventional Low 2021 (*n* = 15 samplings, LED 5.6 lx 3000 K). 3: Tailored Low 2022 (*n* = 16 samplings, LED 5.2 lx 3000 K). Dark controls were sampled simultaneously with the lit treatments. 12 luminaires per treatment. *P* values lower than 0.05 are printed in bold.

#### b) Taxa

To evaluate the influence of individual taxa on the multivariate variability between treatments, we performed Principal Component Analysis (PCA). PCA indicated that the first five principal components together explain 68.8% of the multivariate variance between treatments at the experimental field site in Westhavelland (Fig. 2b, Supplementary Table 3). PC1 (33.6%) was strongest and negatively associated with *Nematocera*, *Lepidoptera*, *Heteroptera*, *Ephemeroptera* and *Trichoptera*. *Odonata*, *Orthoptera* and *Psocoptera* (very low numbers, mainly in dark control) were positively associated. PC2 (12.8%) was negatively associated with *Auchenorrhyncha*, *Psocoptera* and *Thysanoptera* and positively with *Sternorrhyncha*, *Ephemeroptera* and *Brachycera*.

#### c) Community composition

To test for multivariate differences in community composition between treatments, we performed Permutational multivariate analysis of variance (PERMANOVA) based on a multivariate distance matrix of insect taxa (determined to order/suborder level) at individual luminaires in different treatments (groups). This showed significant differences between groups (Fig. 2c, Supplementary Table 4).

#### d) Community dispersion

To test for homogeneity of variances within groups (treatments), we analyzed multivariate homogeneity of group dispersions (variances in species composition) by a pairwise comparison of distances of individual luminaires to group centroids (Tukey post-hoc test). This showed significant differences between treatments at the experimental field site in Westhavelland (Fig. 2d, Supplementary Table 5).

### (ii) Municipal sites

A total of 1553 insects were caught at the three municipal sites in 2022 (Supplementary Table 6). The most abundant taxa were *Nematocera* (465), *Coleoptera* (329) and *Hymenoptera* (319). Comparing total insect numbers between treatments, the conventional lighting attracted approximately twice as many insects compared to the novel tailored and shielded luminaires over the whole sampling period at each municipal site (Table 3, Supplementary Figs. 18–20).

**Table 3:** Total number of attracted insects at three municipal sites

Site	Alter Flugplatz		Backofen Riedwiesen		Heimbachaue	
Treatment	Conventional LED	Tailored LED	Conventional HPS	Tailored LED	Conventional HPS	Tailored LED
Total number of Insects	417	187	300	133	343	173
Factor to Tailored LED	2.2		2.2		1.98	

Alter Flugplatz *n* = 9 samplings, 5 luminaires per treatment, transition from conventional luminaires (LED 4000 K) to tailored and shielded luminaires (LED 4000 K); Backofen Riedwiesen *n* = 7 samplings, 5 luminaires per treatment, transition from conventional luminaires (HPS 2000 K) to tailored and shielded luminaires (LED 2700 K); Heimbachaue *n* = 7 samplings, 4 luminaires per treatment, transition from conventional luminaires (HPS 2000 K) to tailored and shielded luminaires (LED 2700 K).

#### a) Abundance

**Fig. 3:**
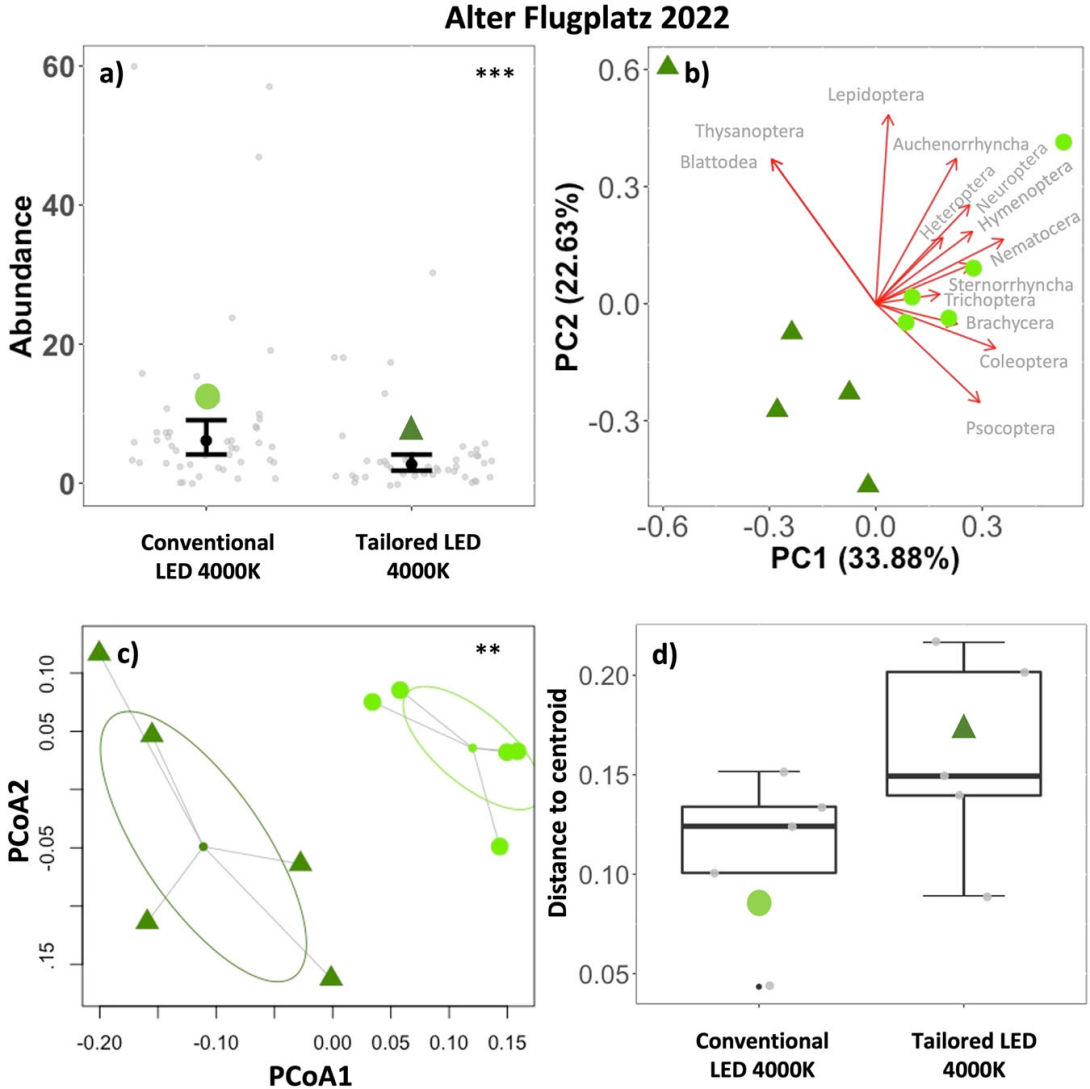
Urban municipal site, Alter Flugplatz, Karlsruhe. **a** Total number of individuals per road light and night (Abundance, gray points) in different treatments (symbols). *n* = 9 samplings, 5 luminaires per treatment, transition from conventional luminaires (LED 4000 K) to tailored and shielded luminaires (LED 4000 K). Predictions (black points) and 95% confidence intervals (error bars) based on a generalized linear regression model (*p* < 0.001***). **b** PCA Principal component analysis of multiple luminaires (*n* = 5) grouped by treatment (symbols). Arrow length represents loadings of individual insect taxa. **c** PERMANOVA Permutational Multivariate Analysis of Variances (df = 1, *R*^***2***^ = 0.387, *F* = 5.058, *p* = 0.007**) of multiple luminaires grouped by treatment (symbols). Ellipses based on SD. **d** Within group dispersion (community dispersion) of luminaires in the same treatment. Distances to centroids (gray points). Boxplots show median (thick line), interquartile range (IQR) (box), outliers (black points), 1.5 × IQR (whiskers), ANOVA (*p* = 0.140).

**Fig. 4:**
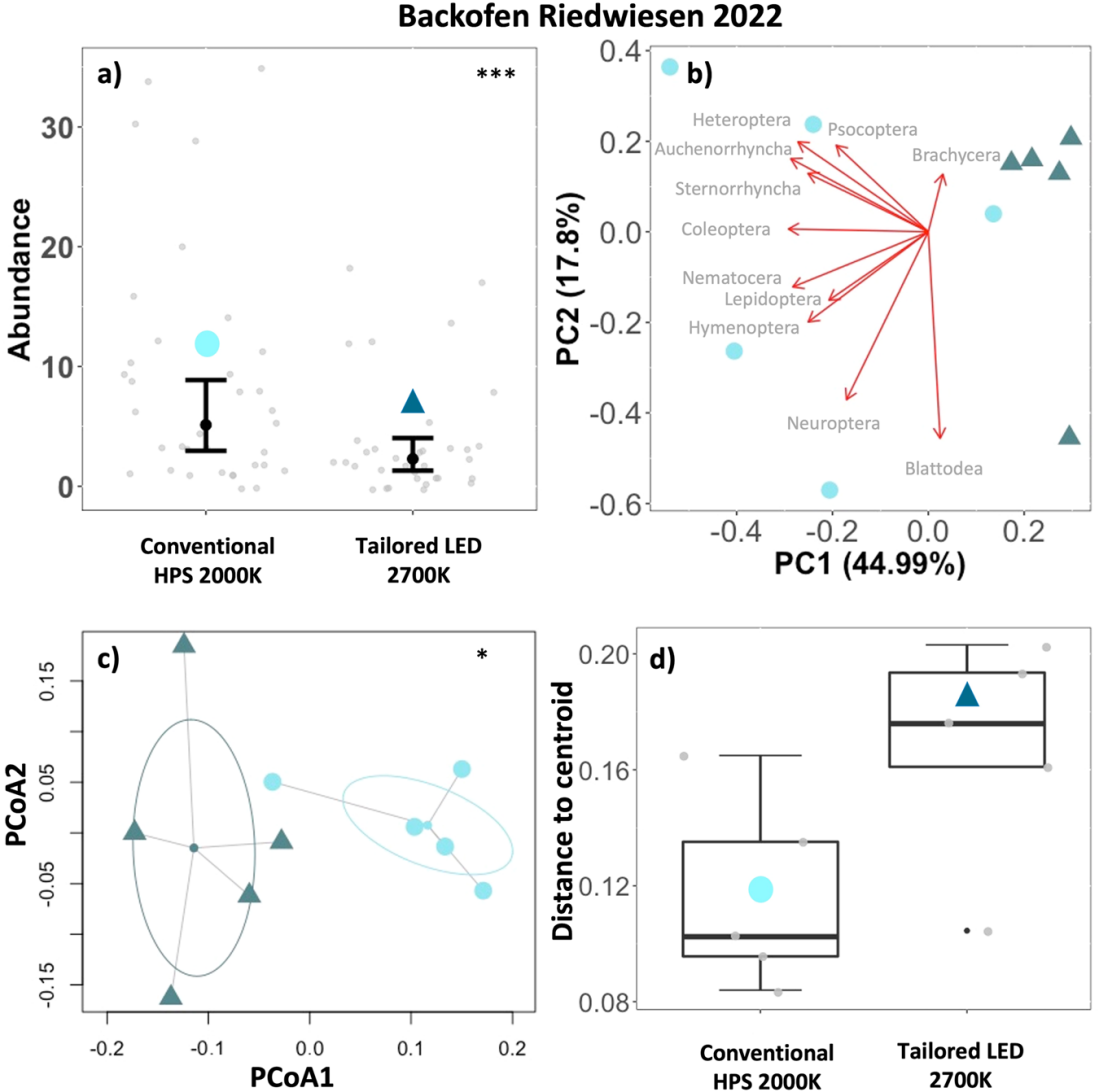
Suburban municipal site, suburban, Backofen Riedwiesen, Brühl. **a** Total number of individuals per road light and night (Abundance, gray points) in different treatments (symbols). *n* = 7 samplings, 5 luminaires per treatment, transition from conventional luminaires (HPS 2000 K) to tailored and shielded luminaires (LED 2700 K). Predictions (black points) and 95% confidence intervals (error bars) based on a generalized linear regression model (*p* < 0.001***) **b** PCA Principal component analysis of multiple luminaires (symbols) grouped by treatment. Arrow length represents loadings of individual insect taxa. **c** PERMANOVA Permutational Multivariate Analysis of Variances (df = 1, *R*^**2**^ = 0.337, *F* = 4.0499, *p* = 0.028*) of multiple luminaires (*n* = 5) grouped by treatment (symbols). Ellipses based on SD. **d** Within group dispersion (community dispersion) of luminaires in the same treatment. Distances to centroids (gray points). Boxplots show median (thick line), interquartile range (IQR) (box), outliers (black points), 1.5 × IQR (whiskers), ANOVA (*p* = 0.055).

**Fig. 5:**
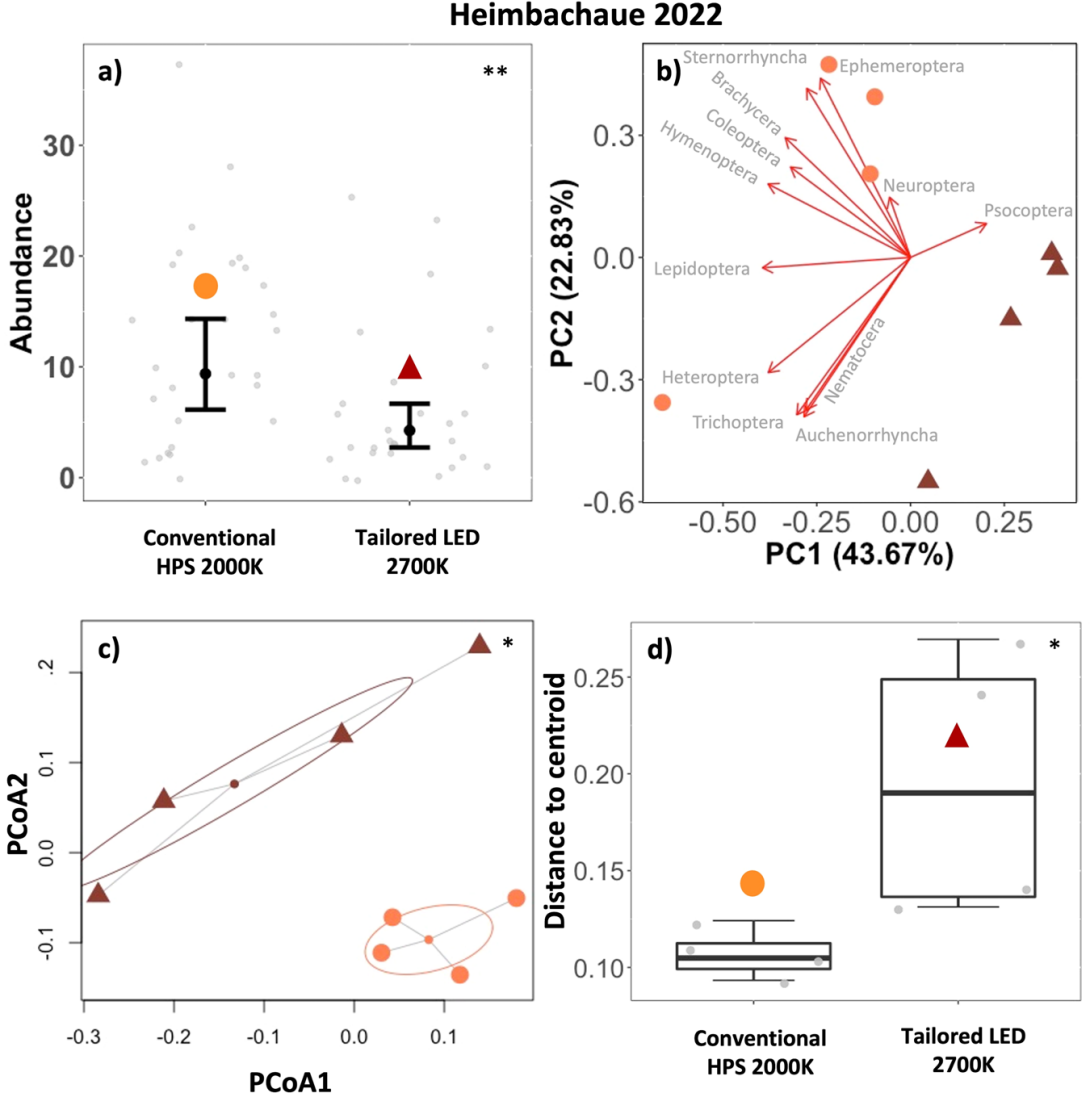
Rural municipal site, rural, Heimbachaue, Betzweiler. **a** Total number of individuals per road light and night (Abundance, gray points) in different treatments (symbols). *n* = 7 samplings × 4 luminaires per treatment, transition from conventional luminaires (HPS 2000K) to tailored and shielded luminaires (LED 2700 K). Predictions (black points) and 95% confidence intervals (error bars) based on a generalized linear regression model (*p* = 0.005**) **b** PCA Principal component analysis of multiple luminaires (*n* = 4) grouped by treatment (symbols). Arrows depict loadings of individual insect taxa. **c** PERMANOVA Permutational Multivariate Analysis of Variances (df = 1, *R*^***2***^ = 0.390, *F* = 3.844, *p* = 0.025 *) of multiple luminaires grouped by treatment (symbols). Ellipses based on SD. **d** Within group dispersion (community dispersion) of luminaires in the same treatment. Distances to centroids (gray points). Boxplots show median (thick line), interquartile range (IQR) (box), outliers (black points), 1.5 x IQR (whiskers), ANOVA (*p* = 0.049*).

Linear regression of the number of insects attracted per light and night (abundance) showed that the novel luminaires attracted significantly less insects than the previously installed conventional luminaires at Alter Flugplatz (Conventional LED vs. Tailored LED 4000 K, *p* < 0.001***), Backofen Riedwiesen (Conventional HPS 2000 K vs. Tailored LED 2700 K, *p* < 0.001***) and Heimbachaue (Conventional HPS 2000 K vs. Tailored LED 2700 K *p* = 0.005**) (Figs. 3a–5a, Table 4). Of the environmental factors, only the temperature had a significant positive effect on insect abundance across treatments at each municipal site (Table 4).

**Table 4:** Effects of light treatments on insect abundance at three municipal sites in Southern Germany (generalized linear model)

Site	abundance ~	Estimate	SE	*Z*	*P*
Alter Flugplatz	Conventional	−0.52241	0.86203	−0.606	0.54450
	Tailored	−0.81214	0.13540	−5.998	2e−09***
	Temperature (°C)	0.18565	0.06137	3.025	0.00248**
	Wind (m/s)	−0.37237	0.25079	−1.485	0.13759
	Precipitation (mm)	−4.81208	10.44150	−0.461	0.64490
Backofen Riedwiesen	Conventional	−0.18780	1.45849	−0.129	0.8975
	Tailored	−0.80635	0.17834	−4.521	6.14e−06***
	Temperature (°C)	0.15150	0.06707	2.259	0.0239*
	Wind (m/s)	−0.32315	0.37422	−0.864	0.3878
	Precipitation (mm)	6.03296	4.23184	1.426	0.1540
Heimbachaue	Conventional	−3.3736	3.7202	−0.907	0.3645
	Tailored	−0.7903	0.2796	−2.827	0.0047**
	Temperature (°C)	0.3519	0.1779	1.978	0.0479*
	Wind (m/s)	0.8018	0.7710	1.040	0.2984
	Precipitation (mm)	−7.1543	4.3940	−1.628	0.1035

Alter Flugplatz *n* = 9 samplings, 5 luminaires per treatment, transition from conventional (LED 4000 K) luminaires to tailored and shielded luminaires (LED 4000 K); Backofen Riedwiesen *n* = 7 samplings, 5 luminaires per treatment, transition from conventional (HPS 2000 K) luminaires to tailored and shielded luminaires (LED 2700 K); Heimbachaue *n* = 7 samplings, 4 luminaires per treatment, transition from conventional (HPS 2000 K) luminaires to tailored and shielded luminaires (LED 2700 K). *P* values lower than 0.05 are printed in bold.

#### b) Taxa

Principal Component analysis showed the association of specific taxa (determined to order/suborder level) with PCs. At the urban site (Alter Flugplatz), in a non-aquatic environment, the first four principal components explain 84.5% of the variance (Fig. 3b, Supplementary Table 7). PC1 (33.9%) was positively associated with *Nematocera*, *Hymenoptera*, *Psocoptera*, *Neuroptera*, *Coleoptera* and *Sternorrhyncha*. *Blattodea*, and *Thysanoptera* (singletons) were negatively related. Lepidoptera were strongly positively associated with PC2 (22.6%).

At the suburban site (Backofen Riedwiesen) the first four principal components explained 85.7% of the variance. PC1 (45.0%) (Fig. 4b, Supplementary Table 8) was negatively related with *Coleoptera*, *Auchenorrhyncha*, *Nematocera*, *Heteroptera*, *Hymenoptera* and *Sternorrhyncha*. *Blattodea* and *Neuroptera* (only 2 individuals in the conventional treatment) were associated with PC2 (17.8%).

At the rural site (Heimbachaue) the first four principal components explained 93.9% of the variance (Fig. 5b, Supplementary Table 9). PC1 (43.7%) was negatively associated with *Hymenoptera*, *Lepidoptera*, *Heteroptera*, *Coleoptera* and *Brachycera* and *Trichoptera*. PC2 (22.8%) was negatively associated with *Nematocera*, *Ephemeroptera*, *Trichoptera*, and *Sternorrhyncha*.

#### c) Community composition

PERMANOVA based on a multivariate distance matrix of insect catches (determined to order/suborder level) at individual luminaires in different treatments (groups) showed significant differences between groups (conventional vs. tailored and shielded luminaires) at all municipal sites (Fig. 3c–5c, Supplementary Table 10).

#### d) Community dispersion

Analysis of multivariate homogeneity of group dispersions (variances in species composition) in individual treatments (groups) by comparison of distances of individual luminaires to group centroids (ANOVA) showed a significant increase in group dispersion in the tailored and shielded treatment (LED 2700 K) compared to the conventional treatment (HPS 2000 K) at Heimbachaue (*p* = 0.049*). The tailored and shielded treatments at Alter Flugplatz (*p* = 0.140) and Backofen Riedwiesen (*p* = 0.055) similarly showed an increase in group dispersion, yet not significant (Fig. 3d–5d, Supplementary Table 11).

To rule out the unlikely case that our results at the sites with conventional HPS (2000 K) were caused by the change in CCT of the new LEDs (2700 K), we performed an additional control experiment at Heimbachaue, setting the CCT of the LEDs also to 2000 K (Supplementary Fig. 21). The results, obtained in five nights, support our conclusions (see Supplementary Figs. 22A–D, 23, Supplementary Tables 12–16).

## Discussion

The results of the first experiment at the controlled experimental field site (i) in the dark sky reserve Westhavelland showed that both conventional treatments (high and low illuminance) attracted significantly more insects than the novel tailored and shielded luminaires (tailored low) or unlit dark control treatments. (Fig. 2a). Surprisingly, dimming of the conventional luminaires by a factor of 5 (conventional high vs conventional low) did not impact insect attraction significantly, thus not supporting our first hypothesis (H1). In contrast, using the novel luminaires (tailored low) reduced insect attraction almost to the level of the dark controls with unlit luminaires (no significant differences). This strongly supports our second hypothesis (H2) and points out the huge potential of a tailored spatial light emission and shielding.

Although assuming a general dose–response relationship in flight-to-light-behavior^24^, the strength of the attraction was therefore more impacted by reducing unintentional light emission via spatial confinement and shielding than by reducing illuminance in the applied range. A further reduction of the light intensity could have led to a measurable reduction effect in terms of insect attraction. However, this is likely to vary between taxa and their associated light sensitivities and makes it challenging to predict a certain response threshold for dimming. In fact, the exact mechanism behind the observed flight-to-light response of many insects is under debate. Fabian et al. ^25^ suggest that at a short range, some insect taxa do not move directly towards the light but tilt their dorsum towards the brightest hemisphere to maintain proper flight control. This can elicit continuous steering around the light. Similarly, Degen et al. ^26^ showed that the flight paths of nocturnal moth species are disturbed by road lighting even at greater distances, but only a small proportion of them ended their flight directly at the light source.

Therefore, it is possible that the actual number of insects caught in traps at the light source represents only a fraction of the insects affected. In any case, tailoring and shielding of the light emission greatly reduces the illuminated area in which insects can get confused, what could explain the observed reduced insect attraction in this treatment.

This positive impact proved to be robust in the second experiment (ii), adapting the tailored and shielded lighting approach to a typical road lighting context in municipalities. The novel luminaires attracted significantly less insects than the previous conventional road lighting at all three municipal sites, from a rural to an urban environment (Figs. 3–5a). In an urban context (Alter Flugplatz), this may be particularly relevant, since a constant nocturnal illumination may have already reduced the local insect fauna or induced behavioral adaptations such as a reduced flight-to-light response in local insect populations^27,28^. Regarding spectral tuning, we observed that the reduction remained present when keeping the spectrum constant at 4000 K between conventional and tailored luminaires at Alter Flugplatz. This supports the results obtained at the controlled experimental field site Westhavelland (i) in a realistic urban lighting context. An increase in CCT from the conventional luminaires to the tailored and shielded luminaires (from HPS with 2000 K to LED with 2700 K, Heimbachaue and Backofen Riedwiesen) did not change the positive impact on insect attraction either in a sub-urban or a rural setting. This is not a matter of course, because a higher CCT is expected to increase insect attraction (compared to lower CCT)^29,30^. These findings make us confident that a transition to tailored and shielded luminaires will lead to a reduction of insect attraction in different environmental contexts and for various conventional lighting technologies. To ensure that the observed effect regarding the conversion of HPS luminaires was not driven by the change in CCT, we performed additional control experiments at Heimbachaue with adjusted CCT of the novel luminaires (LED changed from 2700 K to 2000 K), showing similarly a reduced insect attraction (Supplementary Fig. 22A). However, comparing total numbers of attracted insects, we observed that the reduced attraction of the novel luminaires was more pronounced in the 2000 K configuration (reduction to 50% (2700 K) and 33% (2000 K)) (compare Supplementary Figs. 20, 23). This points to the fact that LEDs in a warm white spectrum of 2000 K could constitute an additional optimization to tailored and shielded luminaires (see also Bolliger et al.^19^).

Principal Component Analysis indicated that a large proportion of the multivariate variability between luminaires can be explained by investigating the first and second principal components. These new axes clearly separated luminaires by treatment, yet they were not readily interpretable and not related to specific taxa (many high loadings). Although a more detailed taxonomic resolution could give further insights, this pattern on order level was similar at experimental and municipal sites (Figs. 2–5b, Supplementary Fig. 22B). We therefore think that the observed community shift between conventional and tailored luminaires is not related to individual taxa or photosensitivities but is rather a result of the observed reduced abundance across taxa. However, the most relevant insect taxa varied according to study site and associated ecosystem types, including taxa with aquatic life stages (*Nematocera*, *Ephemeroptera*, *Trichoptera*) where an aquatic context was given in the immediate vicinity. This suggests that proper spatial alignment and shielding of luminaires near water bodies may be especially relevant to reduce the ecological impact of ALAN across habitat boundaries^31,32^. Furthermore, we observed a shift in community structure of the catches to fewer moths (Lepidoptera) at the novel tailored and shielded luminaires at all sites. Moths are important nocturnal pollinators^4^. It is therefore likely that a reported influence of ALAN on plant reproductive success by affecting plant-pollinator interactions^33–35^ can be reduced. Finally, we believe that a transition to our novel luminaires will profit many different flying insects and many important aspects of ecosystem functioning due to a reduced attraction radius via confinement and shielding of the emitted light and consequently a reduction of distracting or misleading visual cues that potentially affects species far beyond the lit area.

The observed reduction in the number of attracted insects was accompanied by a significant change in community composition of attracted insect taxa between treatments at all sites (Figs. 2–5c, Supplementary Fig. 22C), which supports our third hypothesis (H3).

Although a between-year comparison will always include phenological/environmental differences for multiple insect taxa, the unlit control treatments remained almost constant in terms of community composition (and abundance) at the experimental field site in Westhavelland (i). Here, a large proportion of the multivariate variance (63%) was explained by the applied light treatments. The tailored and shielded luminaires clearly captured a species pool more similar to unlit than to lit luminaires.

At the controlled experimental field site Westhavelland (i), the unlit control luminaires and the applied trapping technique sample the local flying insect community passively, rather randomly and less frequently (compared to lit treatments). This causes an increased group dispersion (variance) in the community compositions of individual unlit luminaires (=community dispersion) without attraction effect within the control treatments (Fig. 2d). We observed the same increase in community dispersion at the novel luminaires (and no significant difference to dark controls), yet less pronounced. This is potentially related to the reduced (not eliminated) attraction radius. Consequently, the results obtained with the tailored and shielded treatment falls between full attraction (conventional treatments) and no attraction (no light, dark control treatments) in terms of community dispersion. In contrast, we observed more insects and a distinct and more homogenous composition of insect taxa in conventional lit treatments, potentially reflecting a light-sensitive species pool.

From the municipal sites (ii), we only found a significant increase in community dispersion at the novel luminaires at Heimbachaue (Figs. 3–5d, Supplementary Fig. 22D). Yet, we observed a general increase at tailored and shielded treatments at all municipal sites. Extending the gained insights from the experimental field with dark controls, we think that this increase in community dispersion would have been significant at all the municipal sites if we would have been allowed to turn off the lights completely to have dark controls (instead of “only” shielding them). However, this was not possible in a real-world traffic and policy situation. In addition, at the municipal sites (ii), many potential cofounding factors (such as competing light sources, differences in skyglow) may have affected insect attraction (compared to the controlled experimental field in a dark environment (i).

In summary, we interpret the fact that the tailored and shielded treatment at the controlled experimental set-up in Westhavelland (i) brings luminaires closer to dark controls in abundance, between-group and within-group community dispersion as strong evidence of the huge potential of a tailored lighting field to reduce the fatal attraction of nocturnal insects. These results were obtained with a narrow light distribution, strictly limited to the target area in a naturally dark environment, therefore setting a highly optimized baseline. Similar patterns of abundance, between- and within-group variance were observed in three municipalities in Southern Germany (ii), while illuminating wider streets as well as edges next to the target area. This was done in respect to safety considerations and strengthens the assumption that reducing spill-light and minimizing visibility of luminaire heads truly changes the attraction effect of road lighting towards dark conditions.

Combining the findings from four sites along a gradient in skyglow, spanning a large area of Germany, the value of a tailored light distribution turned out to be robust in different environmental settings. We therefore recommend tailored and shielded luminaires for mitigation strategies to protect insects. This should be applied most importantly in sensitive areas, for example in the vicinity of nature reserves, at freshwater ecosystems or other areas with high biodiversity. Being in line with EU lighting norms (EN 13201), our study has shown that spatial tailoring of the light field can effectively reduce the attraction effect of road lighting on insects without compromising human safety standards. Still, we want to emphasize that artificial outdoor lighting, even if tailored and shielded, is an intervention with an ecological impact, representing a disturbance and a deviation from naturally dark nights. Nevertheless, this study is to our knowledge the first robust evidence to technologically mitigate the effects of road lighting on insect attraction via spatial confinement from an urban context with high skyglow to almost unpolluted rural areas in a dark sky reserve.

## Material and methods

### Tailored lighting design concept

The main approach of this work is to assess the impact of a spatially tailored light distribution on flying insects. In most conventional lighting installations, not only the target area is illuminated, but a lot of spill light is introduced into adjacent areas (compare Fig. 1b–e). Additionally, in conventional luminaires, the exit point of the radiation at the luminaire produces high luminance and emissions at high angles (i.e., nearly horizontally) that correspond to unwanted spill light (see red arrows in Fig. 1b-e). That is why conventional luminaires are often visible from large distances. Our luminaire design aims to reduce both types of spill light by utilizing both optics and different types of shielding to tailor the spatial light emission pattern of a novel LED prototype (developed with Selux Gmbh, Berlin). The geometry of the target area and the conventional lighting installation were determined for each site. That included the width and length of the illuminated (target) surfaces such as roads and pedestrian pathways as well as the height and distance of the luminaires. Further, the illuminance on the target surface was measured for the existing conventional lighting installation at experimental and municipal sites. Those parameters formed the basis of the tailored individual luminaire design, which was customized for each of the sites. The luminaire design followed a multi-step process. First, a corresponding optics was chosen and tested with standard lighting design software (DIALUX evo 11, DIAL GmbH, Lüdenscheid, Germany). Then, additional shielding was added to the luminaire prototype and tested in the laboratory. The shielding was then fine-tuned to fully meet all criteria before one individually customized version of the luminaires was manufactured for each site. After installation, validation measurements (see below for details) were carried out and the illuminance was adjusted (“dimmed”) to be identical to the existing conventional luminaires. At the experimental field site (i), a hard cut-off reduced the illuminance outside of the target area to very low values. In contrast, we refrained from cutting off light exactly at the transition between path and adjacent area at the municipal sites (ii). To ensure visibility of obstacles beside the area to be illuminated, the cut off was set to include ~1–2 m on each path side, so as not to impair any subjective sense of security. At this point, the prototypes are not fully optimized in energy efficiency, which was sacrificed with respect to shielding.

### (i) Insect monitoring experimental field site Westhavelland

The experiments represent one of the few long-term experimental setups to investigate the effect of ALAN over several generations in a rural area in the nature park Westhavelland, Brandenburg, Germany^21,36^. The nature park extends over 1315 km^2^, containing a natural riparian system with running waters, wide wetlands, and forested areas. 2014, a part of the Naturpark was designated an “International Dark-Sky Reserve” by the association DarkSky International. Two experimental fields (52.690669, 12.454857 lit site; 52.691736, 12.464800 dark control site), ~600 m apart from each other, consisting of managed grasslands next to a drainage ditch and adjoining forest edges in a distance, were selected for the installation of 24 commercial road lights in a 20-m distance matrix. For a mapped description of the study site see Supplementary Fig. 1. Management refers to mowing for hay twice per year between June and October without fertilizer application. On the western site, 12 road lights were lit in a way to replicate different urban road lighting conditions in terms of illuminance and light distribution (Supplementary Fig. 2). On the eastern field, the same lights were constantly unlit, representing a highly standardized control site^36^. To evaluate the attraction effect on nocturnal flying arthropods, all luminaires were equipped with flight interception traps (Supplementary Fig. 3) and monitored on a monthly basis between March and October each year (replicates). For this purpose, containers with 80% alcohol were screwed to the bottom of the traps for the period between sunset and sunrise. To aim for comparable lighting conditions, all sampling nights were scheduled corresponding to decreasing half-moon (around the third quarter). Insect identification was done with a binocular and taxonomic resolution was based on insect orders and common suborders. Photometric measurements of the light situations (mobiLux USB lux meter, Czibula & Grundmann GmbH Berlin, Jeti specbos 1201-UV spectrometer, Jeti Technische Instrumente GmbH Jena) and measurements of light technical characteristics of the luminaires (Goniophotometer LMT Go-DS-2000) were carried out by TU Berlin (Supplementary Table 17). Experiments have been carried out with different light treatments from 2019 to 2022 (Supplementary Table 18). From March 2019 until October 2020, LED luminaires (*E*_m_ = 30.6 lx, 3000 K) were installed in 4.5 m height and monitored over two years. These lights correspond to EU road lighting norm EN 13201 for roads categorized for motorized traffic at medium to high speeds and conflict zones such as intersections with pedestrians. In 2021, the same LED luminaires were dimmed (reduction of 82%*, *E*_m_ = 5.6 lx, 3000 K). This corresponds to an EU P4 norm for restricted traffic areas up to 30 km/h on residential streets, parking lots and cycle paths. To aim for comparable sample sizes, these lights were monitored on two consecutive nights per month over one year. In 2022, the tailored and shielded LED luminaires (*E*_m_ = 5.2 lx, 3000 K) were installed (Supplementary Fig. 4). These luminaires with an external glare shield surrounding the LED panels, particularly reducing the high radiance near the LED panels at angles beyond the lit area, were adjusted to a 2.5-m path width in front of the luminaires and emit light only onto the target area, while fulfilling P4 standards according to the road lightning norm E13201. With a Vaisala weather transmitter WXT520 device, environmental temperature, wind speed, precipitation, were measured automatically on the field site.

### (ii) Insect monitoring municipal sites

**Fig. 6:**
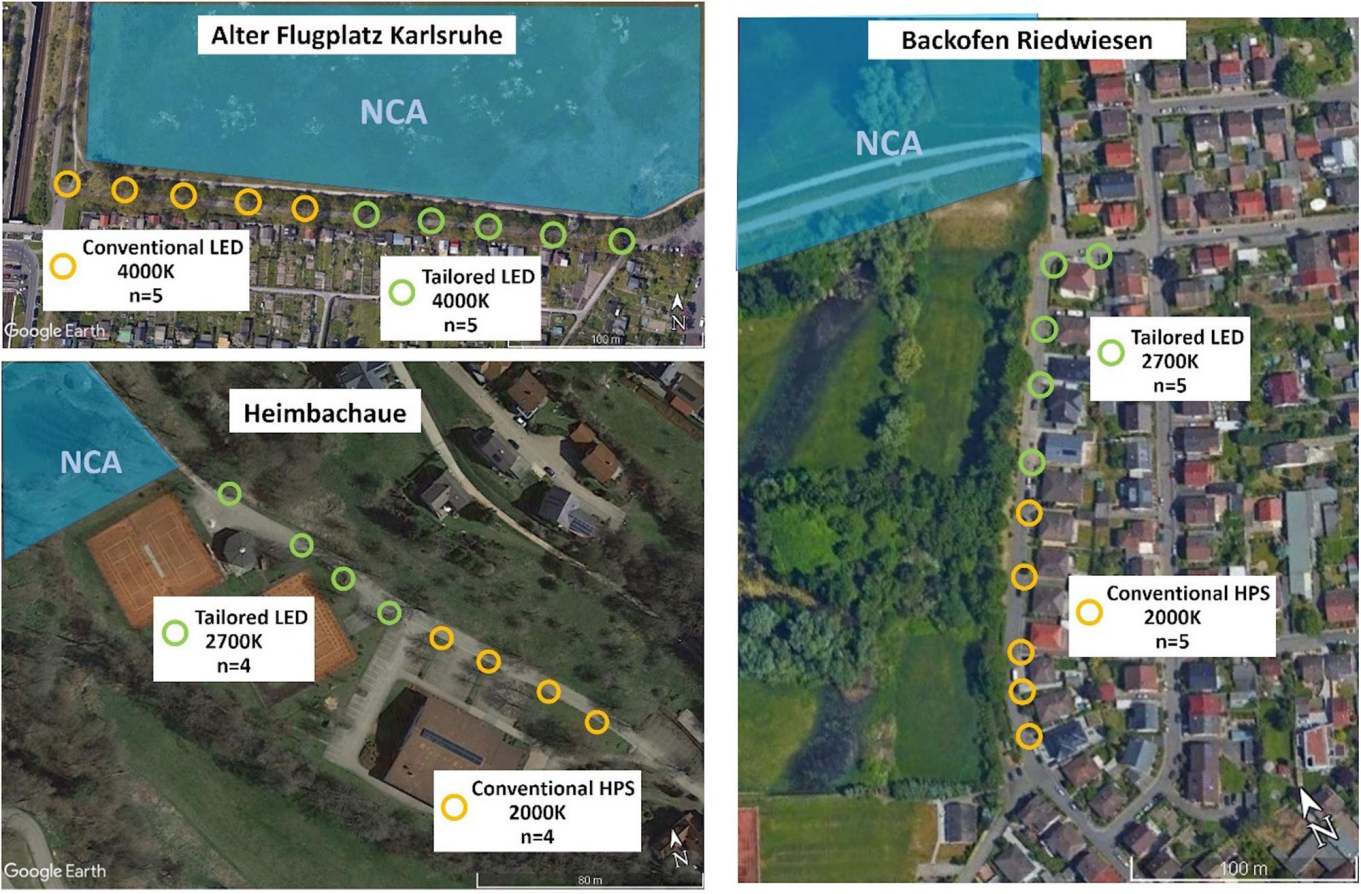
Municipal Sites and selected luminaires for installation of flight interception traps and conversion concept in Baden-Württemberg. The adjacent nature conservation areas are indicated with a bluish area and the letters NCA. Orange circles represent the existing conventional luminaires and green circles represent the luminaires converted to tailored and shielded LED luminaires. Alter Flugplatz *n* = 9 samplings, 5 luminaires per treatment, transition from conventional (LED 4000 K) luminaires to tailored and shielded luminaires (LED 4000 K), Backofen Riedwiesen n = 7 samplings, 5 luminaires per treatment transition from conventional (HPS 2000 K) luminaires to tailored and shielded luminaires (LED 2700 K). Heimbachaue *n* = 7 samplings, 4 luminaires per treatment, transition from conventional (HPS 2000 K) luminaires to tailored and shielded luminaires (LED 2700 K). Maps data: Google, © 2022 CNES/Airbus, Maxar technologies.

We simultaneously installed tailored and shielded luminaires (LED) at streets in three municipalities in 2022. Suitable nature reserves in the administrative region of Karlsruhe within the German federal state Baden-Württemberg were categorized into adjusted pollution classes regarding sky brightness (Supplementary Table 19, Supplementary Fig. 24). To make general statements on the transition to tailored and shielded luminaires, protected areas (*n* = 3) were selected from different habitats, environmental contexts (urban, peri-urban, rural), sky brightness pollution classes and existing lighting structure. However, the main criterion for the site selection consisted in the existence of linear rows of road lights in direct vicinity to the protected areas and the lights being visible from within. Depending on geography and lighting situation, a site-specific monitoring and a conversion concept were developed for the locations (e.g., number of traps, selection of masts/lights, position in relation to the nature reserve, conversion options, traffic usage) (Fig. 6, Supplementary Table 20). To record lighting conditions at the chosen sites, systematic physical measurements using optical measurement technology were carried out at each site (Supplementary Figs. 6–16). A calibrated camera system optimized for nocturnal light measurements was used for this purpose. Measurements were carried out with a sensitive illuminance meter (ILT-1600, International Light Technologies, Peabody, USA) and with spectrometers (JETI Specbos 1211 UV, Jena Technische Instrumente, Jena, Germany). The lighting was characterized in the immediate vicinity of the luminaire, on the street and from different perspectives from the protected area. A DSLR camera (Canon EOS 6D) with two different lenses was used for the luminance measurements. A 50 mm lens with a luminance calibration and the iQ Luminance software (Image Engineering, Kerpen, Germany) was used for small angular ranges or luminance measurements on individual luminaires. A fisheye lens with calibration and software “Sky Quality Camera—SQC” (Euromix, Ljubljana, Slovenia) was used for wide-angle ranges or entire lighting scenes as well as skyglow validation measurements^37^. Additionally, a DJI Mini drone with a built-in camera was used to obtain aerial night-time images. To ensure that the comparison allows reliable statements, control investigations with the existing conventional lighting were carried out at all locations. For these site replicas, the control sections should be as similar as possible (development, vegetation, traffic, lighting environment etc.) to the upgraded sections before the upgrade. One half of the trap-equipped lights were converted to tailored and shielded luminaires, while the other half served as controls (no dark controls in this setup due to legal regulations, not allowing to turn off road lights under real traffic conditions). From April to October 2022, a total of 28 flight interception traps of the same design as in Westhavelland were mounted close to the luminaire heads at three municipal sites. Conventional and tailored treatments were sampled simultaneously on a monthly schedule on consecutive nights close to half-moon (replicates) (Alter Flugplatz *n* = 9 samplings, 5 luminaires per treatment, transition from conventional luminaires (LED 4000 K) to tailored and shielded luminaires (LED 4000 K)*;* Backofen Riedwiesen *n* = 7 samplings, 5 luminaires per treatment, transition from conventional luminaires (HPS 2000 K) to tailored and shielded luminaires (LED 2700 K)*;* Heimbachaue *n* = 7 samplings, 4 luminaires per treatment, transition from conventional luminaires (HPS 2000 K) to tailored and shielded luminaires (LED 2700 K). To rule out that the differences in color temperature between conventional (HPS 2000 K) luminaires and the tailored and shielded luminaires (LED 2700 K) obscures the effect of an optimized light field and affects insect attraction, we additionally performed control experiments with the tailored and shielded luminaires (LED, 2000 K) at Heimbachaue. For this purpose, we attached amber foil over the LED panels inside the luminaire head and increased illuminance to the same level (2000K, *E*_m _= 5.2 lx). From June to September, the tailored and shielded luminaires were modified in this way for the duration of one week after sampling with the original configuration (LED, 2700 K) and prior to five additional sampling events. The experiments at the municipal sites (ii) represent an extension of the experiments at the controlled experimental field site in Westhavelland (i) with the tailored and shielded luminaire prototype and a control site with completely unlit luminaires in the same year. Environmental data for each site (temperature, precipitation, wind) were obtained from the nearest close-by weather station (Alter Flugplatz - Rheinstetten, Backofen Riedwiesen—Mannheim, Heimbachaue—Freudenstadt) of the German weather forecast (open data DWD).

### Site description

#### Alter Flugplatz

The total area of the protected reserve “Alter Flugplatz” is around 70 ha. The entire area lies within the municipal area of Karlsruhe, the third largest in the federal state of Baden-Württemberg. It contains mainly permeable sand substrate, characterized by aridity, a lack of nutrients and large temperature fluctuations. The vegetation is shaped by soil properties and the type of use that was carried out on the airfield in the last decades (including sheep/donkey grazing)^38^. Today, there is a mosaic of sandy grassland communities in different succession stages in the north of the area, transitioning into soil acidic oligotrophic grassland, with interspaced stocks of gray hair grass^38–40^. The South of the area is characterized by open nardus grassland, representing an important habitat for insects (moths, grasshoppers, bees) and bird species^39,40^. If not dominating, this is also true for the woody ruderal vegetation at the southern end (blackberry scrub) along a trail, creating nesting opportunities for songbirds and cavity-nesting insects. In 2009, a species inventory contained 72 species of bees and wasps, 20 grasshopper species, 50 carabid species, 109 spider species and 111 butterfly species, with partly close relationship to foraging plants^38^. Attached to the woody strip in the south is a bicycle track, lined with oaks on the outside of the fence. These trees are mostly covering the road lights under investigation (*n* = 10) from above during the vegetation period, without completely covering visibility from the open grassland (Fig. 6). This is true although road lights face in southern direction, away from the protected area. Distance to nature reserve is ~2 m for all luminaires, distance between luminaires is 30 m. At the southern side of the bicycle track there is an allotment garden area with many different plant species, including non-native species and fruit trees. Regarding sky glow, this site represents the most polluted, brightest municipal site in the experimental setup.

#### Backofen Riedwiesen

The nature reserve at the southern edge of the Rhine-Neckar Metropolitan Region has a size of around 149.3 ha. It is delimited by the Rhine in the west and in the north by Antwerpener Straße and the adjoining industrial area. The road lights and industrial lighting are visible from the whole area and constitute competitive light sources. In the east, the area is limited by the settlement area of Brühl/Rohrhof. In the South, the flood dam and the following agriculturally managed area represent the border. The luminaires under investigation (*n* = 10) are located at Promenadenweg at the eastern end of the dam, facing a small, not agriculturally used area south of the dam, with higher woody vegetation and permanently wet areas with reed (Fig. 6). This patch represents an ecological extension of the actual protected damp meadows north of the dam, although less frequently flooded during flood events in the Rhine. Only the lights at the northern end of the street are directly visible from within the protected area. Distance between luminaires ranges from 17 to 36 m. Regarding sky glow, this site represents the intermediately light polluted municipal site in the experimental setup.

#### Heimbachaue

The nature reserve “Heimbachaue” in Loßburg is located in the municipality of Betzweiler-Wälde in the northern part of black forest. It represents a natural landscape section of the Heimbach-valley between the districts of Betzweiler and Wälde (~1500 citizens). The Heimbach creek flows between predominantly damp valley floors. With fast flowing sections as well as extensive shallow water zones and irregular banks with vegetation of alder-ash floodplain forest, the creek creates a usable habitat for water-bound plants and animals with different requirements^41^. The heart of the nature reserve is located at a former fishpond (a larger and a smaller one). These near-natural water bodies with shallow water zones create a suitable habitat for many insects. On the wood-free eastern valley slope, a typical semi-arid grassland creates different site conditions and causes a small-scale change from very dry to constantly wet biotopes^41^. The western valley slopes are mainly covered with forest (softwoods). In addition, in the wider, rural landscape, large areas are covered with forest. A small street adjoints the southern end, leading to a predominantly sealed area of a large community hall. The available vegetation here is especially represented by multiple interspaced trees, some of them covering the trap-equipped road lights under investigation along the street (Fig. 6). Distance of luminaires ranges from 18 to 33 m. Regarding sky glow, this site represents the most unpolluted, darkest of the municipal sites.

### Statistics and reproducibility

Statistical analysis was done with R Studio Software Version 2022.12.0 + 353. Abundance was calculated counting the total number of individuals in each trap and night in each treatment (Illumination). Abundance analysis (dependant variable) involved generalized mixed linear regression models for each site. Model selection included: distribution tests (“shapiro.test”, ”fitdistr” (“MASS” package^42^), “lmerTest” package^43^ and “DHARMA” package (residual diagnostics for hierarchical (multi-level/mixed) regression models)^44^. Hypothesis testing was carried out using the “anova” function of the “stats” package^45^, backward selection of fixed effects and comparing AIC. “GlmmTMB” models^46^ were generated and the distribution family was set to “nbinom2” for Westhavelland, Alter Flugplatz and Heimbachaue and to “poisson” for Backofen Riedwiesen. To make general statements on the effectiveness of different light treatments, models included the light treatment (factor) as well as environmental predictors mean temperature (°C), mean wind speed (m/s) and mean precipitation (mm) (continuous), expected to be correlated with insect abundance in the traps, as fixed effects and the individual luminaire number and the sampling date as crossed random effects (factor) to account for variability of repeated measures of luminaires across different treatments. All models were checked for overdispersion and zeroinflation. To test for temporal/spatial autocorrelation of residuals for multiple measurements per date/luminaire, scaled residuals were recalculated for each date/luminaire with the “recalculateResiduals” function. Model predictions and confidence intervals were extracted with the “ggemmeans” function of the “ggeffects” package^47^. Post-hoc tests (“emmeans” package, method ”Tukey”^48^) were computed to reveal the differences between individual treatments. Plotting was done with “ggplot2” package^49^. To evaluate species compositions of individual luminaires, we conducted multivariate analyses. Abundances of insect taxa were aggregated by luminaire number and treatment (=Luminaire ID), which resulted in a community matrix for each site. To evaluate the influence of individual insect taxa on compositional differences between luminaires, a Principal Component analysis was performed on the untransformed community matrix with the “prcomp” function (scale=T) and a biplot was used to visualize results. In addition, we plotted the Eigenvalues in a scree plot to determine how many PCs to examine (threshold = 1/number of variables). To understand how each taxon is correlated, we considered taxa with a loading higher than the square of 1/number of variables in our interpretation of the principal components.

Extending PCA with a statistical measure, the resulting community matrix was root squared transformed to minimize the influence of most abundant taxa and a distance matrix using bray-curtis-dissimilarities was generated for PERMANOVA (function “vegdist”, package “vegan”^50^). To test differences in community composition between different treatments (groups) we performed PERMANOVA with the “adonis2” function (permutations = 999, method = “bray”, strata = Luminaire ID). Homogeneity of variances within groups was tested with the “betadispers” function (a multivariate analog to Levene’s test for homogeneity of variances) (group=treatment) and distances to group centroids compared with the “anova” function or pairwise with the “TukeyHSD” function (“stats” package).

### Reporting summary

Further information on research design is available in the Nature Portfolio Reporting Summary linked to this article.

### Supplementary information

Supplementary Material

Reporting Summary

## Data Availability

All data supporting the results is accessible via DOI 10.6084/m9.figshare.23773365. Files names: AuBe_wide_format; AuBe_long_format: The source data behind Fig. 2a-d. NaturLicht_long_format; NaturLicht_wide_format: The source data behind Fig. 3a-d, 4a-d, 5a-d.

## Code availability

All code used in this study is available via 10.6084/m9.figshare.23773365. Files names: Reducing the fatal attraction of nocturnal insects with tailored and shielded road lights.rtf: R-Code Statistical Analysis.
