# Supplementary material for: Reducing the fatal attraction of nocturnal insects using tailored and shielded road lights
Source: Commun Biol. 2024 May 31;7:671. doi: 10.1038/s42003-024-06304-4 (PMC11143364; doi:10.1038/s42003-024-06304-4)
Supplement: Supplementary file 1 — Supplementary Material [file 42003_2024_6304_MOESM1_ESM.pdf]

**Supplementary Information**

Manuscript „Reducing the fatal attraction of nocturnal insects using tailored and shielded road lights“

**Corresponding Author:** Manuel Dietenberger. Leibniz-Institute of Freshwater Ecology and Inland Fisheries, Department 2 Community and Ecosystem Ecology, Light Pollution and Ecophysiology, Müggelseedamm 310, 12587 Berlin, Germany

**E-Mail:** manuel.dietenberger@igb-berlin.de

**Number of**

**Tables:** **20**

**Figures:** **24**

**Supplementary Table 1: Abundance (Individuals) per insect taxon and light treatment at the Experimental field site in Westhavelland from 2019-2022.** Treatment numbers refer to treatments of the same years. 1: Conventional High 2019-2020 (n=14 samplings, LED 30.6 lx 3000K). 2: Conventional Low 2021 (n=15 samplings, LED 5.6 lx 3000K). 3: Tailored Low 2022 (n=16 samplings, LED 5.2 lx 3000K). Dark controls were sampled simultaneously to the lit treatments.

| No. luminaires | 12 | 12 | 12 | 12 | 12 | 12 | |
|---|---|---|---|---|---|---|---|
| **Taxa** | **1. Conventional High** | **2. Conventional Low** | **3. Tailored High** | **1. Dark control** | **2. Dark control** | **3. Dark control** | **TOTAL** |
| **Auchenorrhyncha** | 13 | 0 | 4 | 2 | 1 | 4 | 24 |
| **Blattodea** | 1 | 0 | 0 | 0 | 0 | 1 | 2 |
| **Brachycera** | 30 | 34 | 15 | 14 | 23 | 19 | 135 |
| **Coleoptera** | 292 | 125 | 53 | 193 | 159 | 119 | 941 |
| **Ephemeroptera** | 332 | 369 | 11 | 15 | 7 | 11 | 745 |
| **Heteroptera** | 173 | 33 | 8 | 3 | 4 | 5 | 226 |
| **Hymenoptera** | 55 | 21 | 27 | 20 | 10 | 16 | 149 |
| **Lepidoptera** | 182 | 109 | 51 | 14 | 30 | 8 | 394 |
| **Nematocera** | 2093 | 781 | 322 | 67 | 140 | 125 | 3528 |
| **Neuroptera** | 16 | 9 | 5 | 1 | 1 | 2 | 34 |
| **Odonata** | 0 | 0 | 0 | 0 | 1 | 0 | 1 |
| **Orthoptera** | 0 | 0 | 0 | 0 | 1 | 1 | 2 |
| **Psocoptera** | 7 | 1 | 6 | 17 | 3 | 12 | 46 |
| **Sternorrhyncha** | 29 | 641 | 19 | 18 | 8 | 37 | 752 |
| **Thysanoptera** | 39 | 2 | 42 | 62 | 6 | 59 | 210 |
| **Trichoptera** | 34 | 5 | 3 | 4 | 3 | 2 | 51 |
| **TOTAL** | 3296 | 2130 | 566 | 430 | 397 | 421 | 7240 |

**Supplementary Table 2: Pairwise comparison of light treatments at the Experimental field site in Westhavelland from 2019-2022** (Tukey post hoc test of generalized linear model). Treatment numbers refer to treatments of the same years. 1: Conventional High 2019-2020 (n=14 samplings, LED 30.6 lx 3000K). 2: Conventional Low 2021 (n=15 samplings, LED 5.6 lx 3000K). 3: Tailored Low 2022 (n=16 samplings, LED 5.2 lx 3000K). Dark controls were sampled simultaneously to the lit treatments.

| **contrast** | **Estimate** | **SE** | **Df** | **t-ratio** | **p-value** |
|---|---|---|---|---|---|
| 1. Conventional High- 2. Conventional Low | 0.072 | 0.316 | 1068 | 0.228 | 0.9999 |
| 1. Conventional High- 3. Shielded Low | 1.324 | 0.290 | 1068 | 4.571 | **<0.0001** |
| 1. Conventional High- 1. Dark control | 1.834 | 0.122 | 1068 | 14.975 | **<0.0001** |
| 1. Conventional High- 2. Dark control | 1.435 | 0.330 | 1068 | 4.342 | **0.0002** |
| 1. Conventional High- 3. Dark control | 1.608 | 0.302 | 1068 | 5.325 | **<0.0001** |
| 2. Conventional Low - 3. Tailored Low | 1.252 | 0.305 | 1068 | 4.103 | **0.0006** |
| 2. Conventional Low - 1. Dark control | 1.762 | 0.331 | 1068 | 5.317 | **<0.0001** |
| 2. Conventional Low - 2. Dark control | 1.363 | 0.125 | 1068 | 10.911 | **<0.0001** |
| 2. Conventional Low - 3. Dark control | 1.537 | 0.317 | 1068 | 4.854 | **<0.0001** |
| 3. Tailored Low - 1. Dark control | 0.510 | 0.307 | 1068 | 1.664 | 0.5562 |
| 3. Tailored Low - 2. Dark control | 0.111 | 0.320 | 1068 | 0.346 | 0.9993 |
| 3. Tailored Low - 3. Dark control | 0.285 | 0.125 | 1068 | 2.274 | 0.2055 |
| 1. Dark control - 2. Dark control | -0.399 | 0.325 | 1068 | -1.228 | 0.8233 |
| 1. Dark control - 3. Dark control | -0.225 | 0.296 | 1068 | -0.761 | 0.9739 |
| 2. Dark control - 3. Dark control | 0.174 | 0.310 | 1068 | 0.560 | 0.9935 |

**Supplementary Table 3: Principal component analysis (PCA) at the Experimental field site in Westhavelland.** Values depict loadings of individual insect taxa on Principal components 1-5. Loadings higher than 0.25 were considered important (bold numbers).

| | PC1 | PC2 | PC3 | PC4 | PC5 |
|---|---|---|---|---|---|
| % Variance | 33.58 | 12.84 | 7.84 | 7.75 | 6.82 |
| Auchenorrhyncha | -0.2356213 | **-0.3198239** | 0.07366546 | 0.17283232 | -0.092528 |
| Blattodea | -0.1640521 | -0.1990486 | **-0.3237697** | **0.42059662** | **-0.3026404** |
| Brachycera | -0.1447411 | **0.33382626** | **0.3742818** | **0.25064754** | 0.10752044 |
| Coleoptera | **-0.2690216** | -0.1750551 | 0.24306165 | -0.0293617 | **0.361188** |
| Ephemeroptera | **-0.3500037** | **0.28650078** | 0.06989279 | 0.1257441 | -0.1226591 |
| Heteroptera | **-0.3868926** | -0.0869555 | 0.01027054 | 0.06181093 | 0.01155648 |
| Hymenoptera | -0.2349096 | -0.1087259 | -0.0965189 | -0.3361367 | -0.0053338 |
| Lepidoptera | **-0.3731051** | 0.07394211 | -0.058808 | -0.2079665 | -0.0098119 |
| Nematocera | **-0.4092176** | 0.00825377 | 0.02285197 | -0.0366636 | -0.0091979 |
| Neuroptera | -0.1939558 | 0.08611713 | 0.02469283 | **-0.5608928** | 0.05610538 |
| Odonata | 0.026496 | 0.06369693 | -0.0050283 | **0.36820042** | **0.71083202** |
| Orthoptera | 0.06469994 | -0.0864007 | **0.51009815** | 0.17111427 | -0.4139557 |
| Psocoptera | 0.07502302 | **-0.2893097** | **0.62796759** | -0.1315956 | 0.00512163 |
| Sternorrhyncha | -0.0625816 | **0.56953123** | 0.04036873 | 0.08041811 | -0.2410105 |
| Thysanoptera | 0.04606664 | **-0.3928853** | -0.121507 | 0.02273054 | -0.0673608 |
| Trichoptera | **-0.3688297** | -0.1662645 | -0.0268654 | 0.22039348 | -0.00165 |

**Supplementary Table 4: Permutational multivariate analysis of variance (PERMANOVA)** at the Experimental field site in Westhavelland, showing differences in community composition between treatments (groups).

| ~ treatment | Df | Sumsq | R2 | F | Pr(>F) |
|---|---|---|---|---|---|
| Westhavelland 2019-2022 | 5 | 4.3172 | 0.63297 | 22.764 | 0.001 *** |

**Supplementary Table 5: Pairwise comparison of distances of individual luminaires to group centroids within treatments (groups) at the Experimental field site in Westhavelland (Tukey post hoc test).** Treatment numbers refer to treatments of the same years. 1: Conventional High 2019-2020 (n=14 samplings, LED 30.6 lx 3000K). 2: Conventional Low 2021 (n=15 samplings, LED 5.6 lx 3000K). 3: Tailored Low 2022 (n=16 samplings, LED 5.2 lx 3000K). Dark controls were sampled simultaneously to the lit treatments.

| | difference in observed mean | lower end point Interval | upper end point interval | p-value adjusted |
|---|---|---|---|---|
| 1. Conventional High-2. Conventional low | 0,0059197 | -0,0402532 | 0,05209262 | 0,99897304 |
| 3. Shielded low-2. Conventional low | 0,05212431 | 0,00595139 | 0,09829723 | **0,01796957** |
| 2. Dark control-2. Conventional low | 0,06517572 | 0,01900279 | 0,11134864 | **0,0013467** |
| 3. Dark control-2. Conventional low | 0,07832065 | 0,03214772 | 0,12449357 | **6,95e-05** |
| 1. Dark control-2. Conventional low | 0,09557455 | 0,04940162 | 0,14174747 | **1,01e-06** |
| 3. Shielded low-1. Conventional High | 0,04620461 | 3,17e-05 | 0,09237754 | **0,04974373** |
| 2. Dark control-1. Conventional High | 0,05925602 | 0,0130831 | 0,10542894 | **0,0045947** |
| 3. Dark control-1. Conventional High | 0,07240095 | 0,02622803 | 0,11857387 | **0,00027356** |
| 1. Dark control-1. Conventional High | 0,08965485 | 0,04348193 | 0,13582777 | **4,45e-06** |
| 2. Dark control-3. Shielded low | 0,0130514 | -0,0331215 | 0,05922433 | 0,96093045 |
| 3. Dark control-3. Shielded low | 0,02619634 | -0,0199766 | 0,07236926 | 0,55921912 |
| 1. Dark control-3. Shielded low | 0,04345024 | -0,0027227 | 0,08962316 | 0,07671436 |
| 3. Dark control-2. Dark control | 0,01314493 | -0,033028 | 0,05931785 | 0,95973424 |
| 1. Dark control-2. Dark control | 0,03039883 | -0,0157741 | 0,07657175 | 0,39215465 |
| 1. Dark control-3. Dark control | 0,0172539 | -0,028919 | 0,06342682 | 0,88094672 |

**Supplementary Table 6: Abundance (Individuals) per insect taxon and light treatment at three municipal sites in Southern Germany in 2022.** Alter Flugplatz n=9 samplings, 5 luminaires per treatment, transition from conventional (LED 4000K) luminaires to tailored and shielded luminaires (LED 4000K), Backofen Riedwiesen n=7 samplings, 5 luminaires per treatment transition from conventional (HPS 2000K cylinder) luminaires to tailored and shielded luminaires (LED 2700K), Heimbachaue n=7 samplings, 4 luminaires per treatment, transition from conventional (HPS 2000K bow-shaped) luminaires to tailored and shielded luminaires (LED 2700K).

| Site | Alter Flugplatz | | Backofen Riedwiesen | | Heimbachaue | | |
|---|---|---|---|---|---|---|---|
| Samplings | 9 | | 7 | | 7 | | |
| No. Luminaires | 5 | 5 | 5 | 5 | 4 | 4 | |
| **Taxa** | **Conventional** | **Tailored** | **Conventional** | **Tailored** | **Conventional** | **Tailored** | **TOTAL** |
| **Auchenorrhyncha** | 9 | 3 | 19 | 8 | 4 | 3 | 46 |
| **Blattodea** | 0 | 1 | 1 | 1 | 0 | 0 | 3 |
| **Brachycera** | 6 | 5 | 3 | 6 | 14 | 5 | 39 |
| **Coleoptera** | 138 | 80 | 58 | 24 | 23 | 6 | 329 |
| **Ephemeroptera** | 0 | 0 | 0 | 0 | 16 | 2 | 18 |
| **Heteroptera** | 32 | 13 | 34 | 18 | 12 | 5 | 114 |
| **Hymenoptera** | 132 | 42 | 69 | 32 | 37 | 7 | 319 |
| **Lepidoptera** | 14 | 9 | 13 | 6 | 20 | 9 | 71 |
| **Nematocera** | 66 | 24 | 82 | 32 | 144 | 117 | 465 |
| **Neuroptera** | 1 | 0 | 2 | 0 | 1 | 0 | 4 |
| **Psocoptera** | 9 | 6 | 5 | 3 | 2 | 3 | 28 |
| **Sternorrhyncha** | 8 | 3 | 14 | 3 | 62 | 11 | 101 |
| **Thysanoptera** | 0 | 1 | 0 | 0 | 0 | 0 | 1 |
| **Trichoptera** | 2 | 0 | 0 | 0 | 8 | 5 | 15 |
| **TOTAL** | 417 | 187 | 300 | 133 | 343 | 173 | 1553 |

**Supplementary Table 7: Principal component analysis (PCA) Municipal Site Alter Flugplatz.** Values depict loadings of individual insect taxa on Principal components 1-4. Loadings higher than 0.28 were considered important (bold numbers).

| | PC1 | PC2 | PC3 | PC4 |
|---|---|---|---|---|
| % Variance | 33.88 | 22.63 | 17.17 | 10.79 |
| Auchenorrhyncha | 0.24050021 | **0.39399515** | 0.27342009 | -0.2121215 |
| Blattodea | **-0.3107529** | **0.39202243** | -0.0079875 | -0.2134744 |
| Brachycera | 0.24301752 | -0.0549095 | **0.52206907** | -0.0371794 |
| Coleoptera | **0.35745541** | -0.1209738 | -0.1694928 | -0.1215668 |
| Heteroptera | 0.20000882 | 0.17886973 | -0.2880691 | **-0.4322331** |
| Hymenoptera | **0.28862556** | 0.19569722 | 0.17024063 | 0.19012289 |
| Lepidoptera | 0.03759118 | **0.51277394** | -0.194478 | 0.21776739 |
| Nematocera | **0.3811234** | 0.17485694 | **-0.2813523** | -0.0299031 |
| Neuroptera | **0.28027568** | 0.26835256 | **0.36612882** | -0.0693733 |
| Psocoptera | **0.31029142** | -0.268166 | 0.00933669 | **-0.4586825** |
| Sternorrhyncha | **0.28439092** | 0.10646866 | -0.0815506 | **0.60073465** |
| Thysanoptera | **-0.3107529** | **0.39202243** | -0.0079875 | -0.2134744 |
| Trichoptera | 0.19105812 | 0.02625703 | **-0.5040788** | -0.0181004 |

**Supplementary Table 8: Principal component analysis (PCA) Municipal Site Backofen Riedwiesen.** Values depict loadings of individual insect taxa on Principal components 1-4. Loadings higher than 0.3 were considered important (bold numbers).

| | PC1 | PC2 | PC3 | PC4 |
|---|---|---|---|---|
| % Variance | 44.99 | 17.80 | 12.34 | 10.59 |
| Auchenorrhyncha | **-0.3840247** | 0.2162944 | -0.0994781 | -0.1637348 |
| Blattodea | 0.03309901 | **-0.6105821** | 0.23948746 | 0.17154773 |
| Brachycera | 0.04067656 | 0.17073111 | **0.48278678** | **-0.7039311** |
| Coleoptera | **-0.3904951** | 0.00829872 | 0.10297233 | **0.30976987** |
| Heteroptera | -0.364239 | 0.26645463 | 0.21696604 | 0.06249668 |
| Hymenoptera | **-0.3354182** | -0.2662175 | -0.2042861 | **-0.4510643** |
| Lepidoptera | -0.2777459 | -0.2020255 | **0.31520762** | -0.0741505 |
| Nematocera | **-0.3783892** | -0.1629815 | -0.0695112 | 0.16625742 |
| Neuroptera | -0.228847 | **-0.4964897** | -0.1881449 | -0.2283531 |
| Psocoptera | -0.2575337 | 0.25603234 | **-0.5433436** | -0.0788156 |
| Sternorrhyncha | **-0.3362995** | 0.17287735 | **0.40675309** | 0.23113691 |

**Supplementary Table 9: Principal component analysis (PCA) Municipal Site Heimbachaue.** Values depict loadings of individual insect taxa on Principal components 1-4. Loadings higher than 0.29 were considered important (bold numbers).

| | PC1 | PC2 | PC3 | PC4 |
|---|---|---|---|---|
| % Variance | 43.67 | 22.86 | 18.08 | 9.29 |
| Auchenorrhyncha | -0.2723873 | -0.3755749 | -0.0263781 | **-0.4199659** |
| Brachycera | **-0.3204839** | 0.28247124 | 0.08123023 | **-0.359479** |
| Coleoptera | **-0.3070527** | 0.21310789 | -0.069098 | **0.36493297** |
| Ephemeroptera | -0.2302752 | **0.42212777** | **0.31645239** | 0.02114359 |
| Heteroptera | **-0.3639887** | -0.2712249 | -0.0140519 | 0.21450567 |
| Hymenoptera | **-0.3645556** | 0.17310792 | **-0.3042132** | -0.0853762 |
| Lepidoptera | **-0.3786662** | -0.0242929 | -0.1990551 | **0.31222869** |
| Nematocera | -0.267004 | **-0.3590159** | 0.0712376 | **-0.4417162** |
| Neuroptera | -0.0537008 | 0.14183897 | **-0.6219865** | -0.227393 |
| Psocoptera | 0.19464541 | 0.07988313 | **-0.5925921** | 0.01731835 |
| Sternorrhyncha | -0.2651799 | **0.39820853** | 0.10708702 | -0.117901 |
| Trichoptera | **-0.2909096** | **-0.3707629** | -0.0291216 | **0.38596696** |

**Supplementary Table 10: Permutational multivariate analysis of variance (PERMANOVA)** at three Municipal Sites, showing differences in community composition between treatments (groups).

| ~ treatment | Df | Sumsq | R2 | F | Pr(>F) |
|---|---|---|---|---|---|
| Alter Flugplatz 2022 | 1 | 0.13000 | 0.38735 | 5.058 | 0.007 ** |
| Backofen Riedwiesen 2022 | 1 | 0.11069 | 0.33609 | 4.0499 | 0.028 * |
| Heimbachaue 2022 | 1 | 0.13685 | 0.39048 | 3.8438 | 0.025 * |

**Supplementary Table 11: Comparison of distances of individual luminaires to group centroids within treatments (groups) for three Municipal Sites** in Southern Germany (ANOVA).

| **Distances to centroid ~ treatment** | **Df** | **Sumsq** | **Mean Sq** | **F** | **Pr(>F)** |
|---|---|---|---|---|---|
| Alter Flugplatz 2022 | 1 | 0.005879833 | 0.005879833 | 2.687716 | 0.1397561 |
| Backofen Riedwiesen 2022 | 1 | 0.006552443 | 0.006552443 | 5.033116 | 0.05512696 |
| Heimbachaue 2022 | 1 | 0.015639222 | 0.015639222 | 6.032961 | 0.04937285 |

**Supplementary Table 12: Abundance (Individuals) per insect taxon and light treatment at Heimbachaue in 2022. Values refer to control experiments with conventional and tailored luminaires at the same CCT (2000K).** n=5 samplings between July and October 2022, 4 luminaires per treatment, transition from conventional luminaires (HPS 2000K bow-shaped) to tailored and shielded luminaires (LED 2000K).

| Order | Conventional HPS 2000K | Tailored LED 2000K | TOTAL |
|---|---|---|---|
| Auchenorrhyncha | 8 | 2 | 10 |
| Brachycera | 10 | 0 | 10 |
| Coleoptera | 11 | 4 | 15 |
| Ephemeroptera | 4 | 0 | 4 |
| Heteroptera | 4 | 3 | 7 |
| Hymenoptera | 33 | 9 | 42 |
| Lepidoptera | 17 | 13 | 30 |
| Nematocera | 164 | 51 | 215 |
| Psocoptera | 3 | 3 | 6 |
| Sternorrhyncha | 16 | 5 | 21 |
| Trichoptera | 5 | 2 | 7 |
| TOTAL | 275 | 92 | 367 |

Factor Conventional to Tailored: 2.99

**Supplementary Table 13: Effects of light treatments on insect abundance at Heimbachaue (generalized linear model). Results refer to control experiments with conventional and tailored luminaires at the same CCT (Conventional HPS 2000K bow-shaped vs. Tailored LED 2000K).** n=5 samplings between July and October 2022, 4 luminaires per treatment, transition from conventional luminaires (HPS 2000K bow-shaped) to tailored and shielded luminaires (LED 2000K). Model structure: abundance ~ Luminaire + mean temperature + mean wind speed + (1|trap_number) + (1|starting_date), family=nbinom1)

| Site | abundance ~ | Estimate | SE | Z | P |
|---|---|---|---|---|---|
| Heimbachaue | Conventional | 6.3989 | 2.52115 | 2.538 | 0.011146 * |
| | Tailored | -1.0867 | 0.20716 | -5.246 | **1.56e-07** *** |
| | Temperature (°C) | -0.0092 | 0.13100 | -0.070 | 0. 943891 |
| | Wind (m/s) | -1.2718 | 0.37240 | -3.415 | **0.000637** *** |

**Supplementary Table 14: Principal component analysis (PCA) Municipal Site Heimbachaue. Results refer to control experiments with conventional and tailored luminaires at the same CCT (Conventional HPS 2000K bow-shaped vs. Tailored LED 2000K).** n=5 samplings between July and October 2022, 4 luminaires per treatment, transition from conventional luminaires (HPS 2000K bow-shaped) to tailored and shielded luminaires (LED 2000K). Values depict loadings of individual insect taxa on Principal components 1-4. Loadings higher than 0.30 were considered important (bold numbers).

| | PC1 | PC2 | PC3 | PC4 |
|---|---|---|---|---|
| **Auchenorrhyncha** | **-0.36000835** | -0.14786840 | 0.21876540 | **0.441738202** |
| **Brachycera** | **-0.39138819** | 0.02677569 | **-0.39111556** | -0.216960934 |
| **Coleoptera** | -0.29158503 | **-0.41999020** | -0.15004296 | -0.162294911 |
| **Ephemeroptera** | -0.29524787 | 0.18859199 | **-0.54044850** | 0.004851440 |
| **Heteroptera** | -0.18336714 | **0.33289843** | -0.04151374 | **0.677916497** |
| **Hymenoptera** | -0.28788880 | 0.24647803 | 0.14587287 | -0.310485809 |
| **Lepidoptera** | -0.23309991 | 0.00981428 | **0.57986172** | -0.075859296 |
| **Nematocera** | **-0.45363421** | 0.02550339 | -0.05898345 | 0.086033033 |
| **Psocoptera** | -0.05866727 | **-0.57163427** | -0.06102385 | -0.049794624 |
| **Sternorrhyncha** | **-0.34697813** | -0.27407950 | 0.26815649 | 0.008075842 |
| **Trichoptera** | -0.21108632 | **0.43732649** | 0.21498856 | -0.399698494 |

**Supplementary Table 15: Permutational multivariate analysis of variance (PERMANOVA) Municipal Site Heimbachaue. Results refer to control experiments with conventional and tailored luminaires at the same CCT (Conventional HPS 2000K bow-shaped vs. Tailored LED 2000K)**, showing differences in community composition between treatments (groups). n=5 samplings between July and October 2022, 4 luminaires per treatment, transition from conventional luminaires (HPS 2000K bow-shaped) to tailored and shielded luminaires (LED 2000K).

| ~ treatment | Df | Sumsq | R2 | F | Pr(>F) |
|---|---|---|---|---|---|
| Heimbachaue 2022 | 1 | 0. 5234681 | 0.39048 | 6. 590974 | 0. 031* |

**Supplementary Table 16: Comparison of distances of individual luminaires to group centroids (ANOVA) within treatments (groups) at Heimbachaue. Results refer to control experiments with conventional and tailored luminaires at the same CCT (Conventional HPS 2000K bow-shaped vs. Tailored LED 2000K).** n=5 samplings between July and October 2022, 4 luminaires per treatment, transition from conventional luminaires (HPS 2000K bow-shaped) to tailored and shielded luminaires (LED 2000K).

| Distances to centroid ~ treatment | Df | Sumsq | Mean Sq | F | Pr(>F) |
|---|---|---|---|---|---|
| Heimbachaue 2022 | 1 | 0. 009531838 | 0.015639222 | 6. 310328 | 0.04577703* |

**Supplementary Table 17: Light Characteristics of light treatments at the Experimental field site in Westhavelland.** Conventional Low 2021 (LED, 5.6 lx, 3000K), Tailored Low 2022 (LED, 5.2 lx, 3000K).

| **Parameter** | **unit** | **Conventional Low** | **Tailored Low** |
|---|---|---|---|
| luminous flux | lm | 691,2 | 310 |
| dimming | % | 16 | 52 |
| power | W | 8,224 | 19,552 |
| light output | lm/W | 84,05 | 15,86 |
| Upper Light Output Ratio ULOR | | | |
| Imax >= 70° | cd/klm | 357 | 33,5 |
| Imax >= 80° | cd/klm | 141 | 3,39 |
| Imax >= 90° | cd/klm | 0 | 0 |
| Treshold Increment TI | % | 7 | 0 |
| Edge Illuminance Ratio EIR | | 0,64 | 0,05 |
| luminaire distance | m | 20 | 20 |
| luminairehight | m | 4,85 | 4,85 |
| street width | m | 2,5 | 2,5 |
| luminaire arm | m | 0,4 | 0,4 |
| luminaire tilt | ° | 0 | 10° |
| Em path | lx | 5,6 | 5,18 |
| Emin | lx | 1,2 | 1,06 |
| Uo | | 0,21 | 0,20 |
| Edge area | | | |
| Emax,-2,5m | lx | 2,6 | 0,09 |
| Emin,-2,5m | lx | 0,43 | 0,04 |
| Emax,+2,5m | lx | 2,1 | 0,13 |
| Emin,+2,5m. | lx | 1,2 | 0,1 |

**Supplementary Table 18: Characterization of applied light treatments from 2019 to 2022 at the Experimental field site in Westhavelland.**

| Treatment | Year | Luminaire | Illuminant | No | Spectrum (K) | Mean lux (Em) | Min lux (Emin) | Sampling events |
|---|---|---|---|---|---|---|---|---|
| 1. Conventional high | 2019 | Schreder Teceo | LED | 12 | 3000 | 30.6 | 8.5 | 7 |
| 1. Conventional high | 2020 | Schreder Teceo | LED | 12 | 3000 | 30.6 | 8.5 | 8 |
| 2. Conventional low | 2021 | Schreder Teceo | LED | 12 | 3000 | 5.6 | 1.2 | 15 |
| 3. Tailored low | 2022 | Selux Tal + shield | LED | 12 | 3000 | 5.2 | 1.2 | 16 |

**Supplementary Table 19: Classification scale of protected areas in Baden-Württemberg according to sky brightness.** Luminance obtained with an all-sky camera and processed with Sky Quality Camera software (see Material & Methods). Values were categorized in terms of zenith luminance.

| Site | Sky brightness (mcd/ $m^2$) | Pollution class |
| --- | --- | --- |
| Westhavelland | 0.23 (Milky Way substracted) | Very low |
| Alter Flugplatz | 2.4 | extreme |
| Backofen Riedwiesen | 1.6 | very high |
| Heimbachaue | 0.29 | low |

**Supplementary Table 20: Classification of light treatments and sampling design at three municipal sites in Baden Württemberg, Southern Germany.**

| | Alter Flugplatz. Karlsruhe | Backofen Riedwiesen. Brühl | Heimbachaue. Betzweiler |
|---|---|---|---|
| **Coordinates** | 49.02107968828992. 8.37779407517844 | 49.41185086276461. 8.519436152709618 | 48.363683514004585. 8.481609229369308 |
| **Habitat** | Dry and sandy oligotrophic grasslands | Damp meadows, Wet floodplains | Wet Floodplain vegetation, Semi-arid grassland |
| **Traffic usage** | Cycle path. No motorized vehicles. pedestrians | Residential street <30km/h. Motorized vehicles + sidewalk | marketplace + parking lot. Slow Motorized vehicles |
| **Pollution class** | Extreme | Very high | Low |
| **Light regime** | Urban | Peri-urban | Rural |
| **Luminaire Height (m)** | 4.5 | 3.5 | 3.5 |
| **No. luminaires per treatment** | 5 | 5 | 4 |
| **Sample events** | 9 | 7 | 7 |
| **Conventional treatment** | | | |
| **Luminaire** | Trilux Lumega 700 | Abele Geiger Trend | Hess Barcelona |
| **Illuminant** | LED | HPS | HPS |
| **Mean Illuminance (lux)** | 9.5 | 3.1 | 5.1 |
| **CCT (K)** | 4000 | 2000 | 2000 |
| **Tailored and shielded treatment** | | | |
| **Luminaire** | Selux Tal + shield | Selux Tal + shield | Selux Beta + shield |
| **Illuminant** | LED | LED | LED |
| **Mean illuminance (lux)** | 9.2 | 3.1 | 5.2 |
| **CCT (K)** | 4000 | 2700 | 2700 |

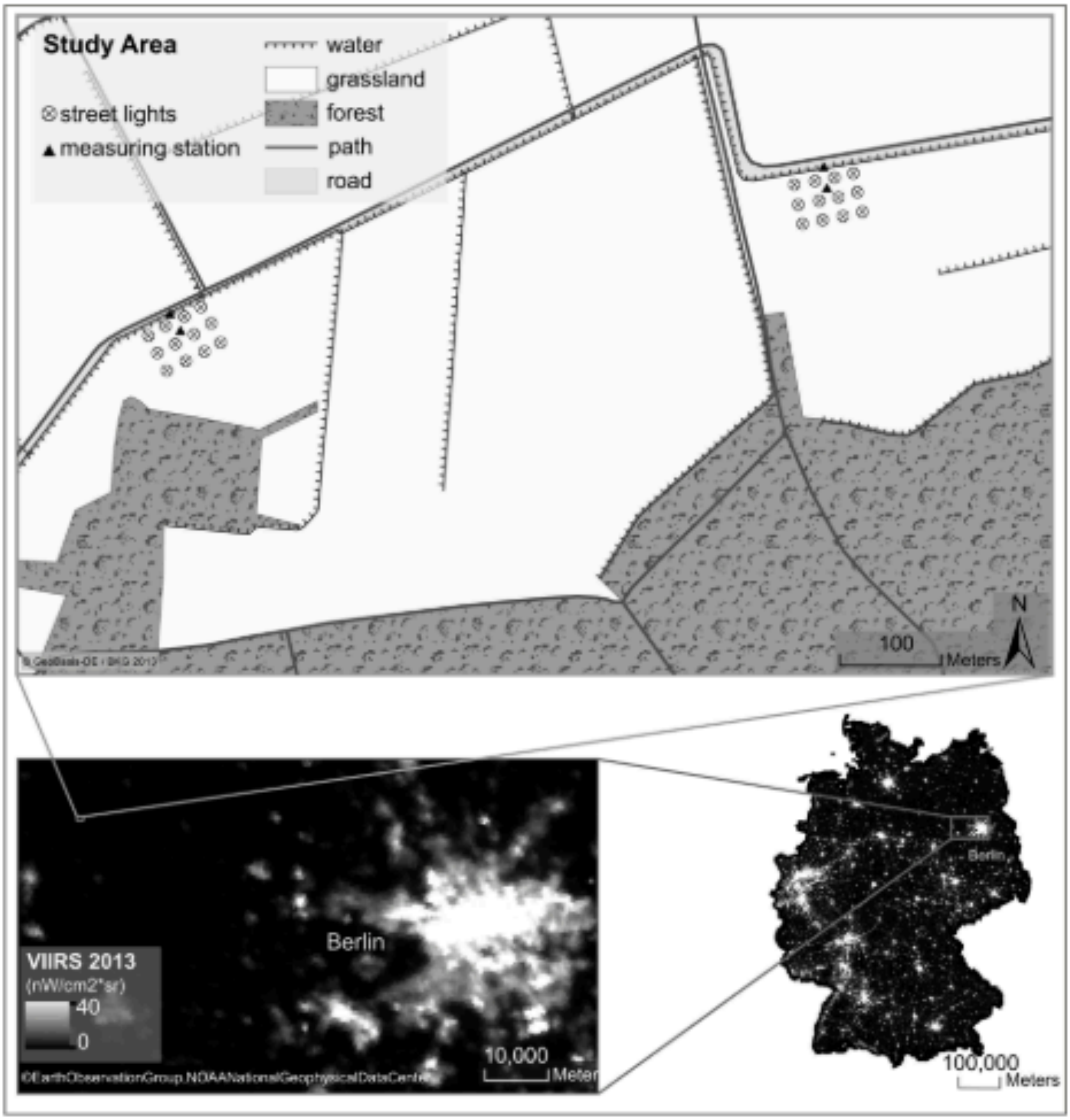


**Supplementary Figure 1: Experimental field site Westhavelland** derived from Holzhauer et al. 2015[1]. Western field: Lit treatments, n=12 luminaires, Eastern field: dark control treatment, n=12 luminaires.

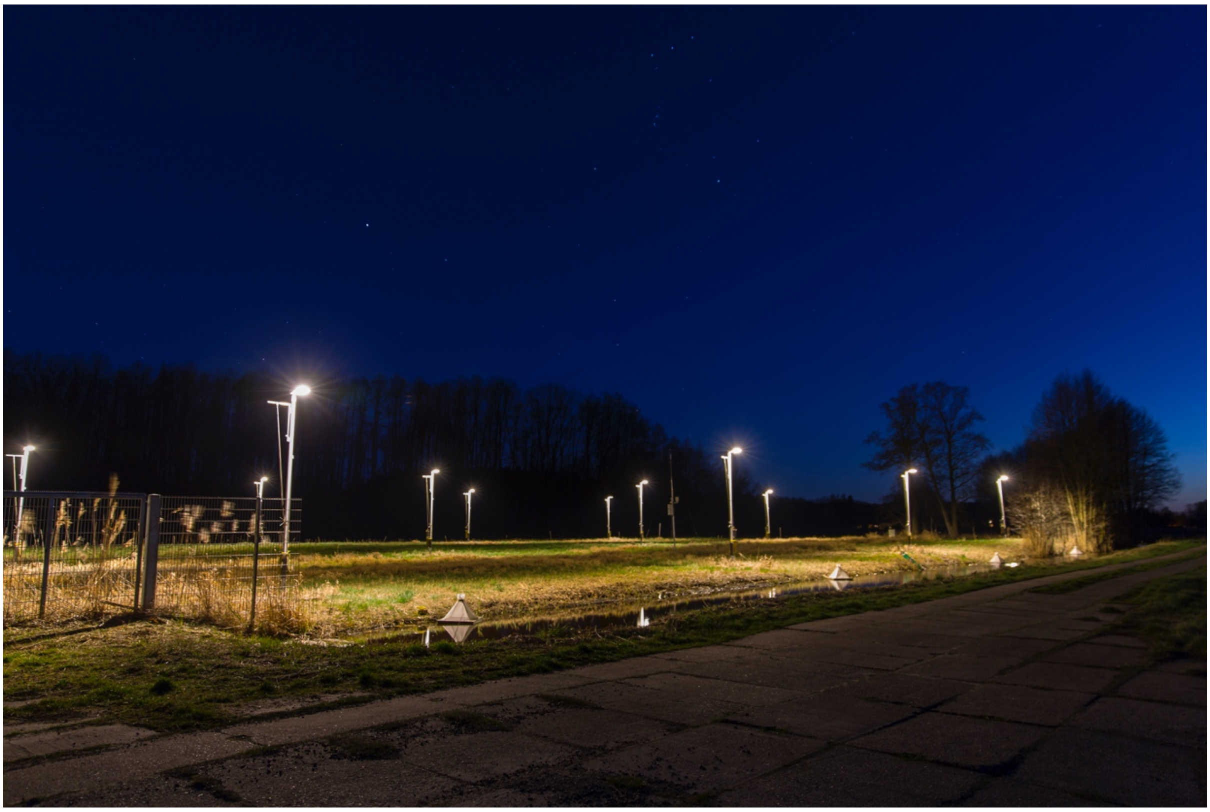

**Supplementary Figure 2: Undimmed Schreder Teceo luminaires (LED, 30.6 lux, CCT 3000K)** installed from 2019-2020 on the western field of the Experimental site in Westhavelland. n=12 luminaires.

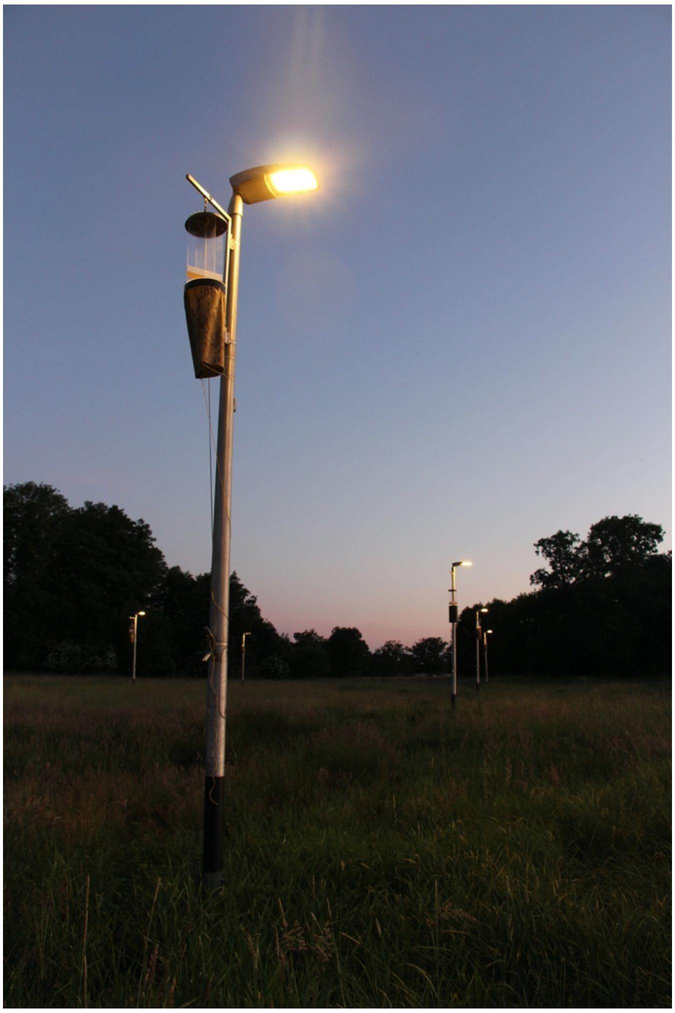

**Supplementary Figure 3: Schreder Teceo LED luminaires equipped with flight interception traps** on the western field of the Experimental site in Westhavelland. n=12 luminaires.

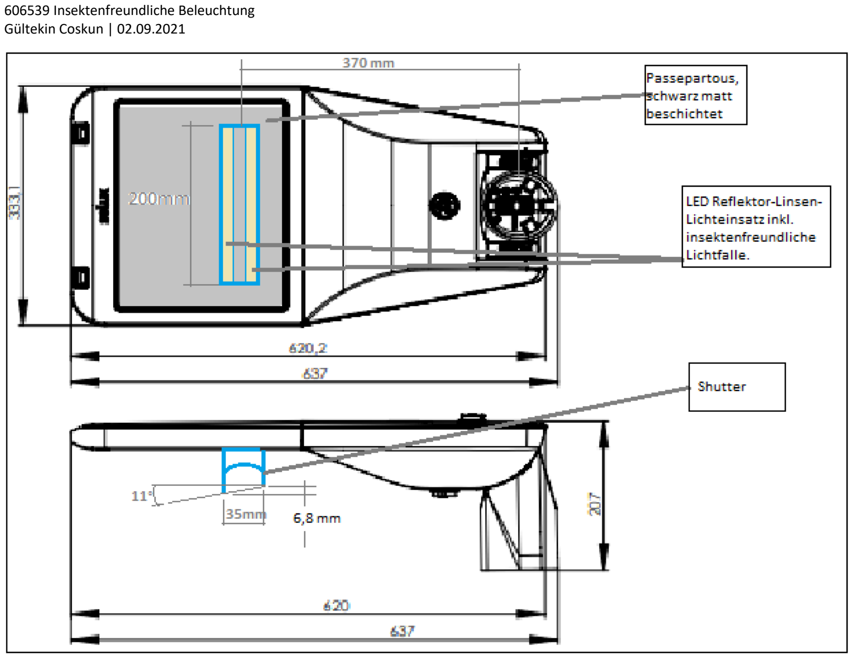


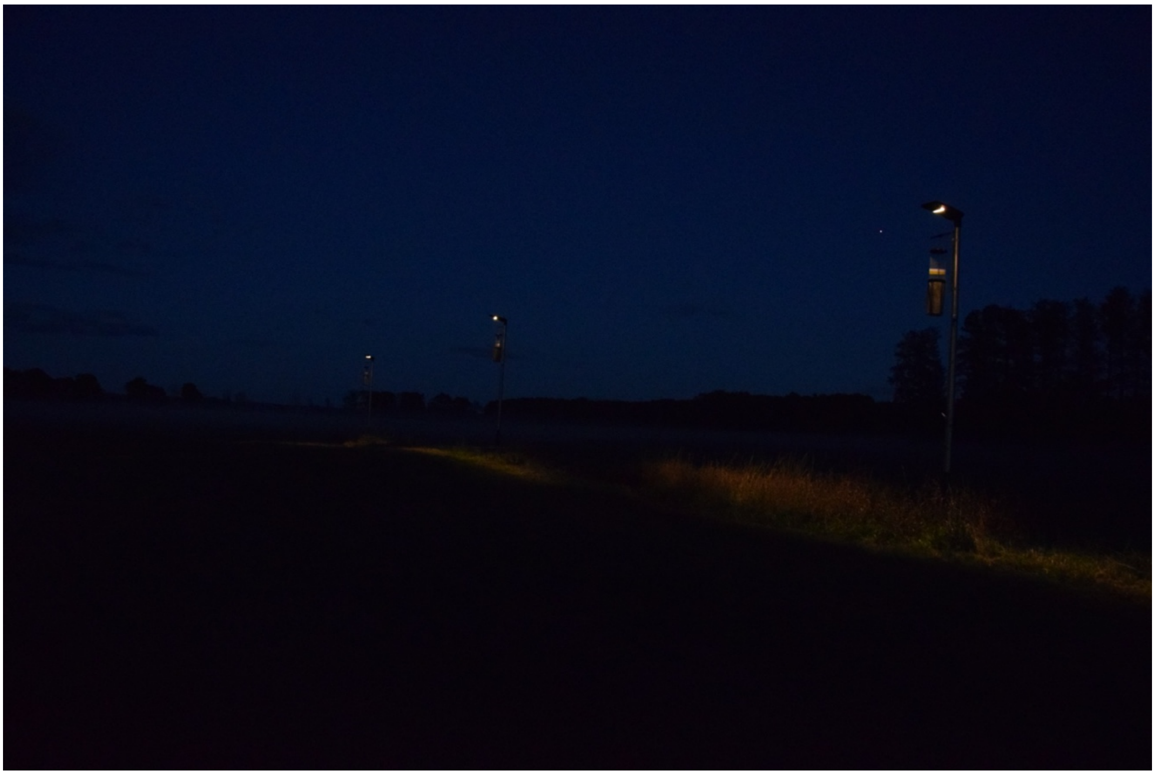

**Supplementary Figure 4:** Schematic drawing of the tailored and shielded SELUX prototype with external shield © Selux. Coskun Gültekin (top), application at the western field of the Experimental site in Westhavelland (bottom). n=12 luminaires.

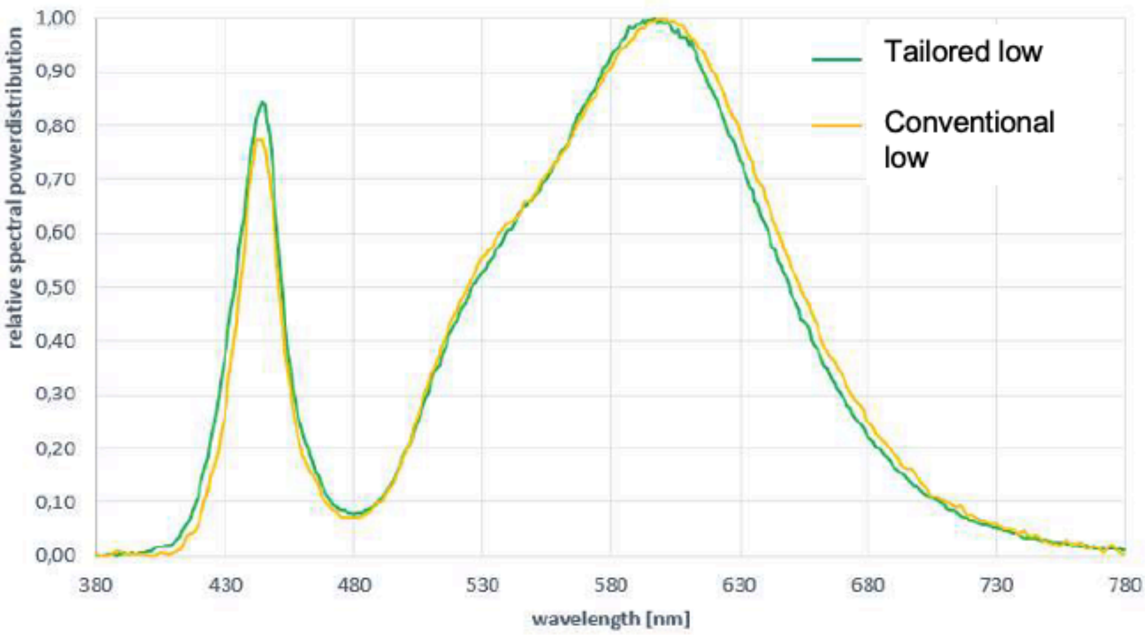


**Supplementary Figure 5: Spectra of luminaires on the western field of the Experimental site in Westhavelland.** Conventional low 2021: second treatment, Schreder Teceo luminaires (LED, $E_m$=5.6 lx, CCT 3000K). Tailored low 2022: third treatment, Selux Tal (LED, $E_m$=5,2 lx, CCT 3000K).

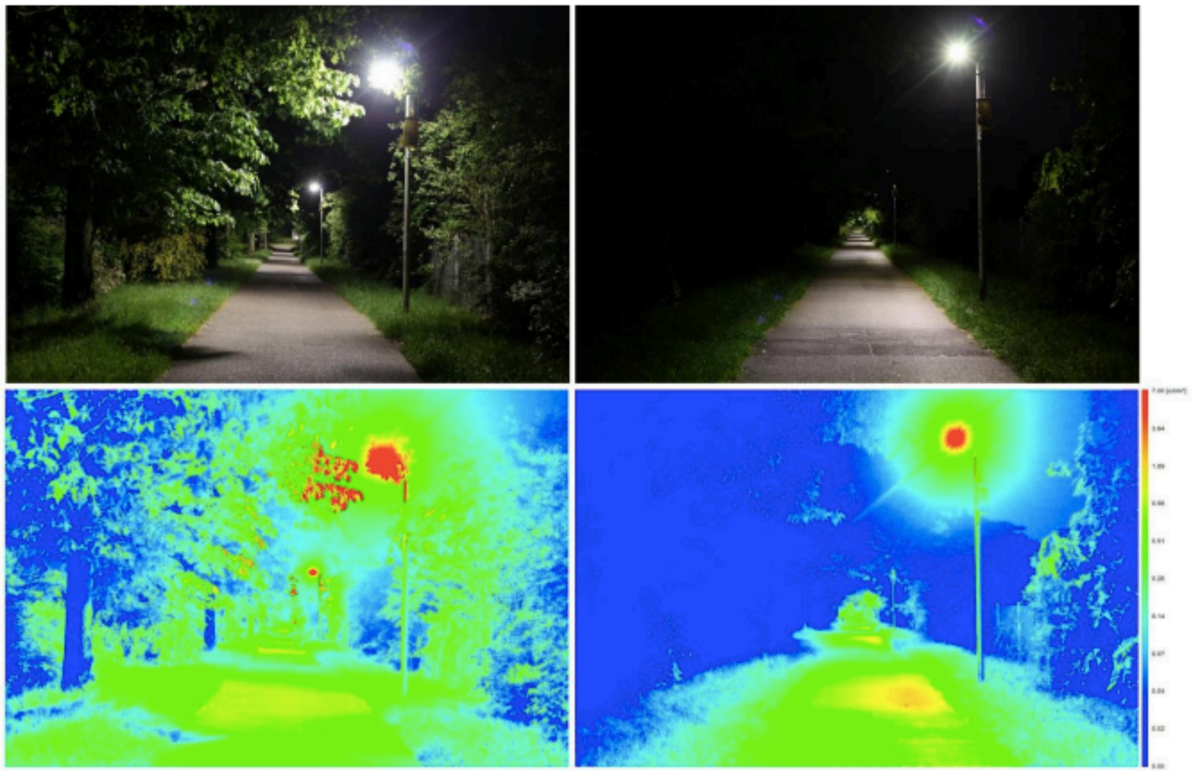

**Supplementary Figure 6: Luminance measurements of luminaires at the Municipal Site Alter Flugplatz.** Conventional luminaires (LED, $E_m$ = 9.5 lx, CCT 4000K) (left) and tailored and shielded luminaires (LED, $E_m$ = 9.2 lx, CCT 4000K) (right), n=9 samplings, 5 luminaires per treatment.

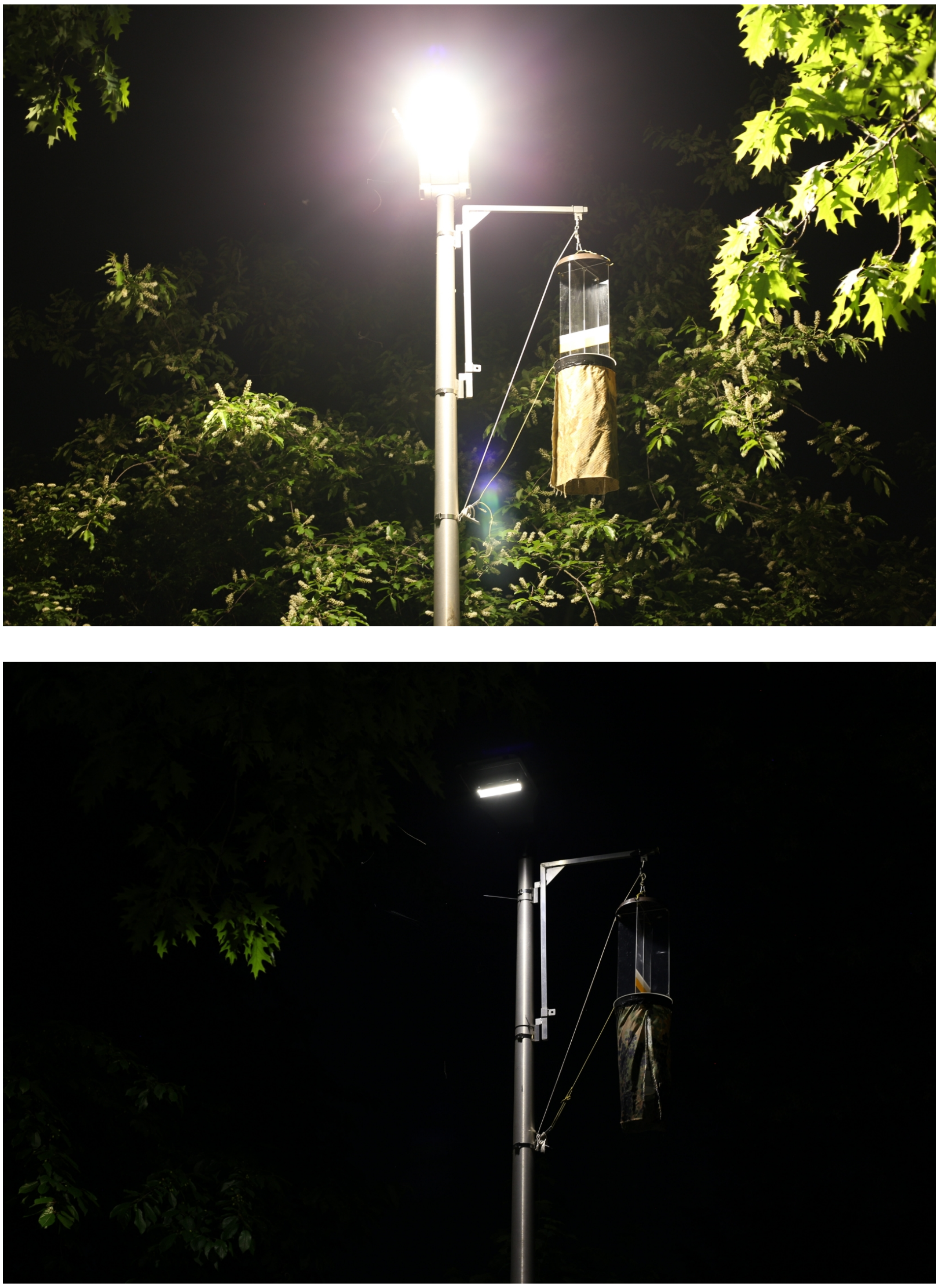

**Supplementary Figure 7: Road lights at the Municipal Site Alter Flugplatz.** Photos were taken at the same distance to luminaires. Conventional luminaires (LED, $E_m$ = 9.5 lx, CCT 4000K) (top) and tailored and shielded luminaires (LED, $E_m$ = 9.2 lx, CCT 4000K) (bottom). n=9 samplings, 5 luminaires per treatment.

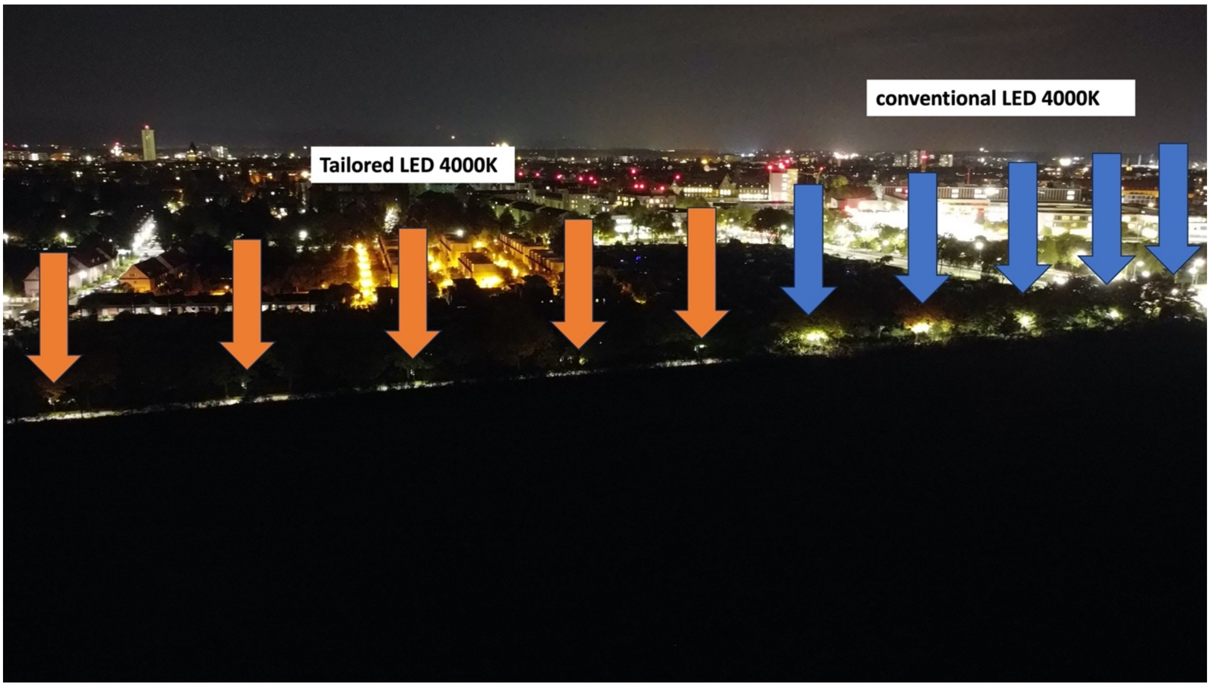


**Supplementary Figure 8: Road lights at the Municipal Site Alter Flugplatz.** Tailored and shielded luminaires (LED, $E_m$ = 9.2 lx, CCT 4000K), n=5 (orange arrows). Conventional luminaires (LED, $E_m$ = 9.5 lx, CCT 4000K), n=5 (blue arrows). Photos captured by drone from within the nature reserve.

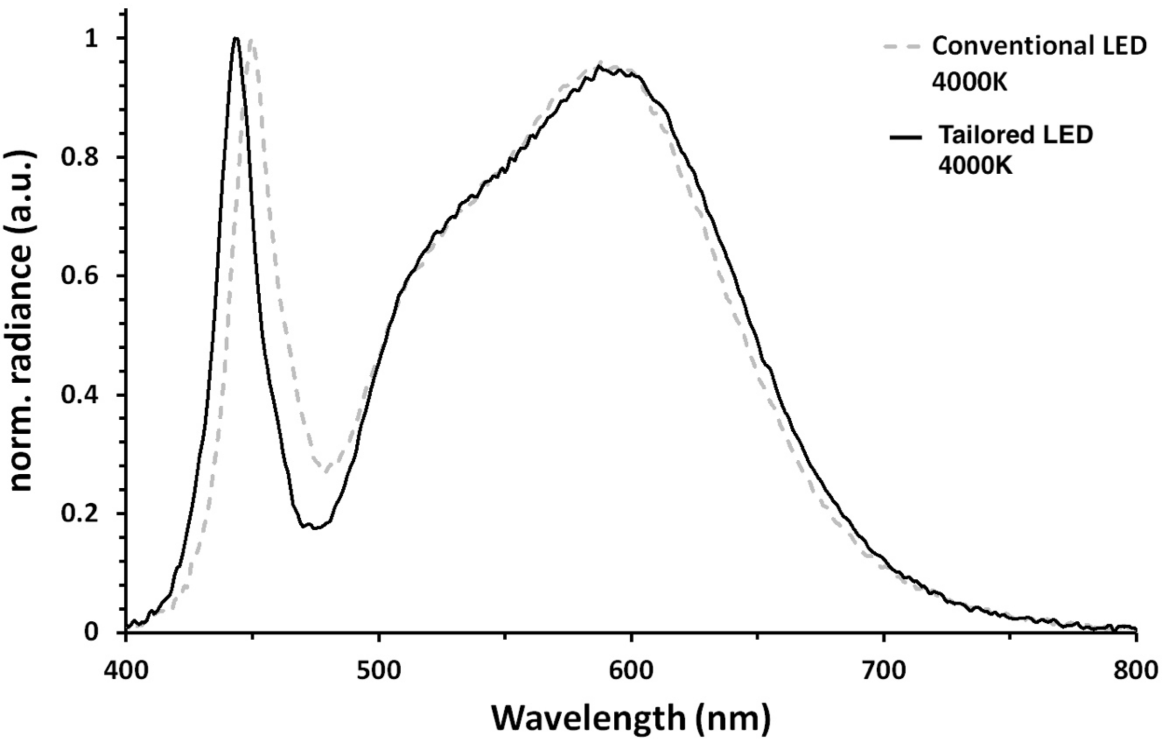


**Supplementary Figure 9: Spectra of luminaires at the Municipal Site Alter Flugplatz.**

Tailored and shielded luminaires (LED, $E_m$ = 9.2 lx, CCT 4000K). Conventional luminaires (LED, $E_m$ = 9.5 lx, CCT 4000K).

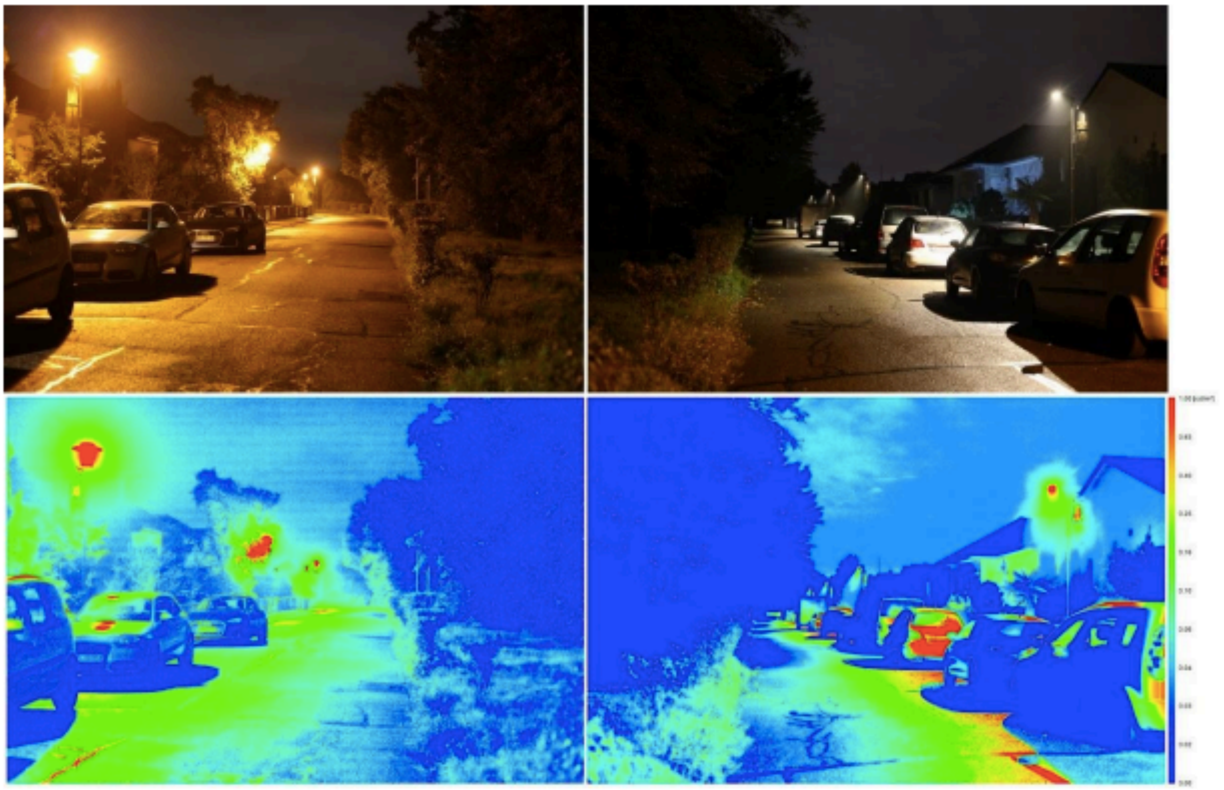

**Supplementary Figure 10: Luminance measurements of luminaires at the Municipal Site Backofen Riedwiesen.** Conventional luminaires (HPS cylinder, $E_m$ = 3.1 lx, CCT 2000K) (left) and tailored and shielded luminaires (LED, $E_m$ = 3.1 lx, CCT 2700K) (right). n=7 samplings, 5 luminaires per treatment.

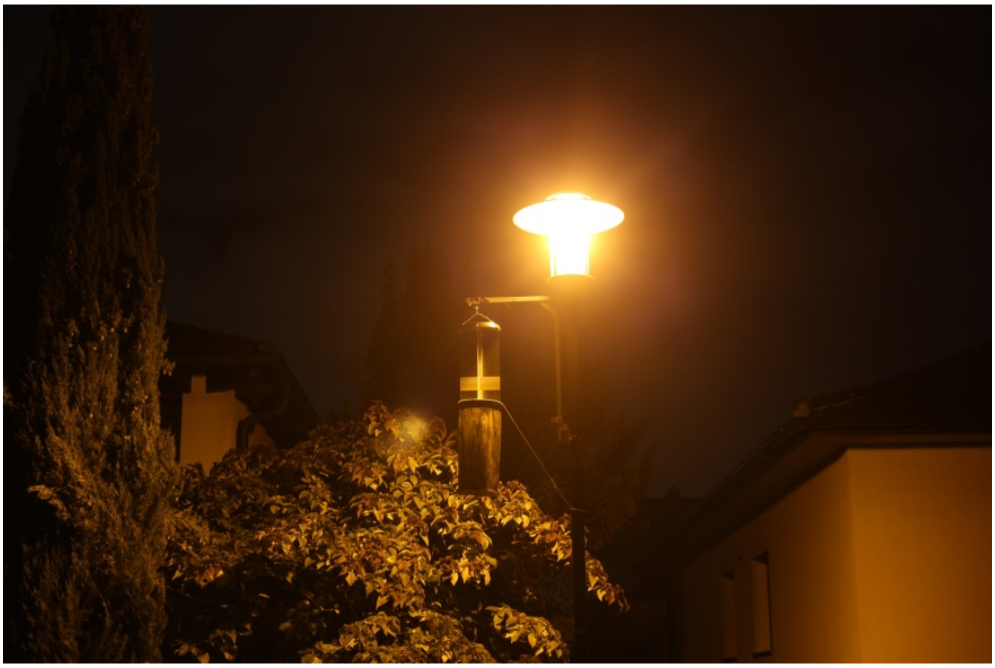

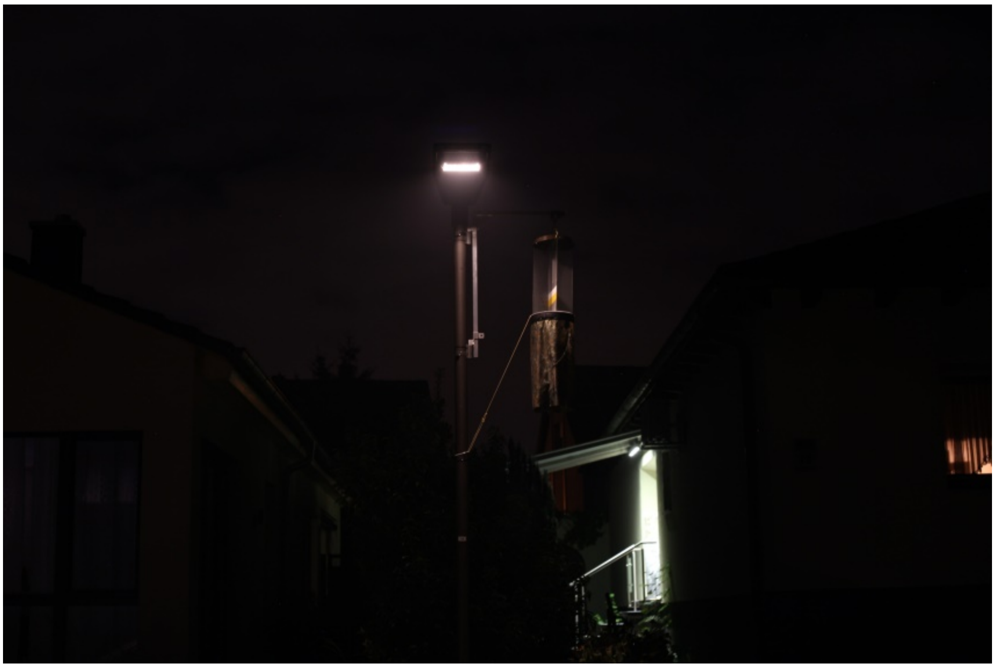

**Supplementary Figure 11: Road lights at the Municipal Site Backofen Riedwiesen.** Photos were taken from the same distance to luminaires. Conventional luminaires (HPS cylinder, $E_m$ = 3.1 lx, CCT 2000K) (top) and tailored and shielded LED luminaires ($E_m$ = 3.1 lx, CCT 2700K) (bottom). n=7 samplings, 5 luminaires per treatment.

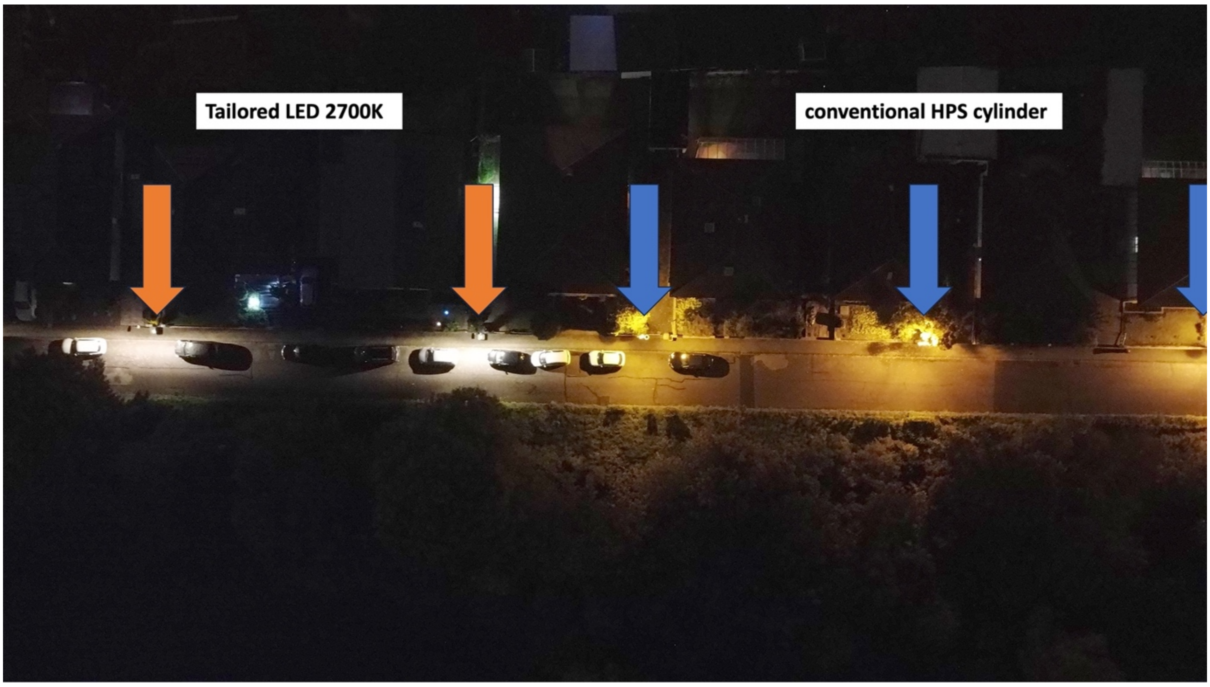


**Supplementary Figure 12: Road lights at the Municipal Site Backofen Riedwiesen.**

Tailored and shielded luminaires (LED, $E_m$ = 3.1 lx, CCT 2700K) n=5 (orange arrows). Conventional luminaires (HPS cylinder, $E_m$ = 3.1 lx, CCT 2000K) n=5 (blue arrows). Photos captured by drone.

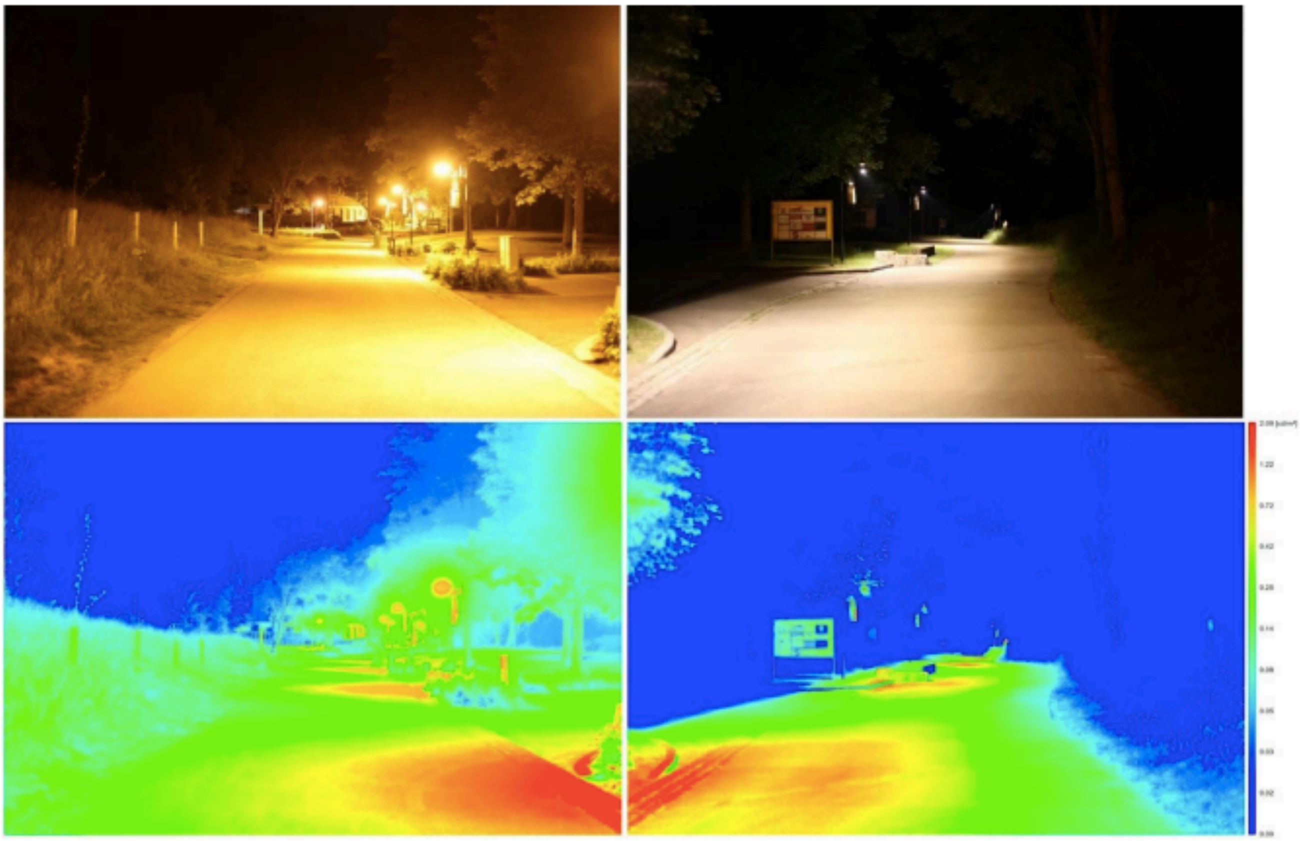

**Supplementary Figure 13: Luminance measurements of luminaires at the Municipal Site Heimbachaue.** Conventional luminaires (HPS bow-shaped, $E_m$ = 5.1 lx, CCT 2000K) (left) and tailored and shielded luminaires (LED, $E_m$ = 5.1 lx, CCT 2700K) (right). n=7 samplings, 4 luminaires per treatment.

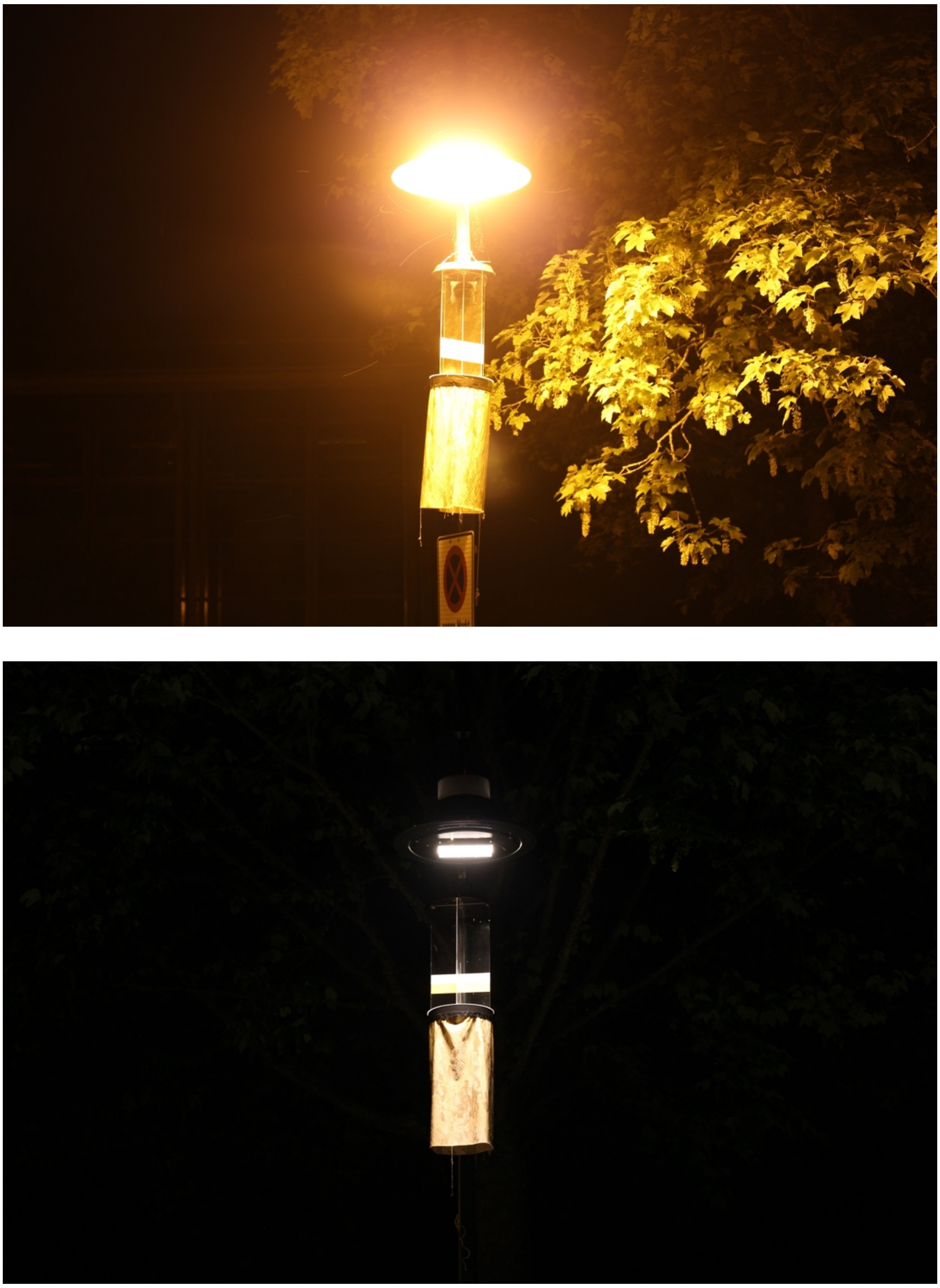

**Supplementary Figure 14: Road lights at the Municipal Site Heimbachaue.** Photos were taken from the same distance to luminaire. Conventional luminaires (HPS bow-shaped, $E_m$ = 5.1 lx, CCT 2000K) (top) and tailored and shielded luminaires (LED, $E_m$ = 5.1 lx, CCT 2700K) (bottom). n=7 samplings, 4 luminaires per treatment.

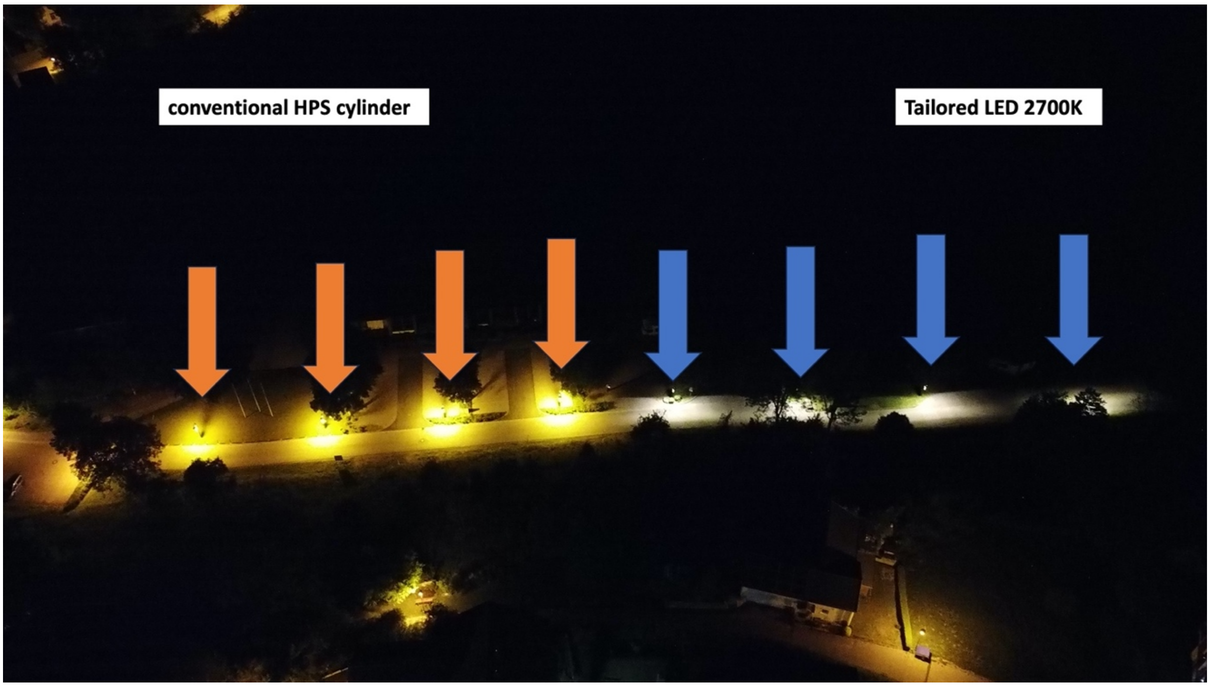


**Supplementary Figure 15: Road lights at the Municipal Site Heimbachaue.** Tailored and shielded luminaires (LED, $E_m$ = 5.1 lx, CCT 2700K) n=4 (blue arrows). Conventional luminaires (HPS bow-shaped, $E_m$ = 5.1 lx, 2000K) n=4 (orange arrows). Photos captured by drone.

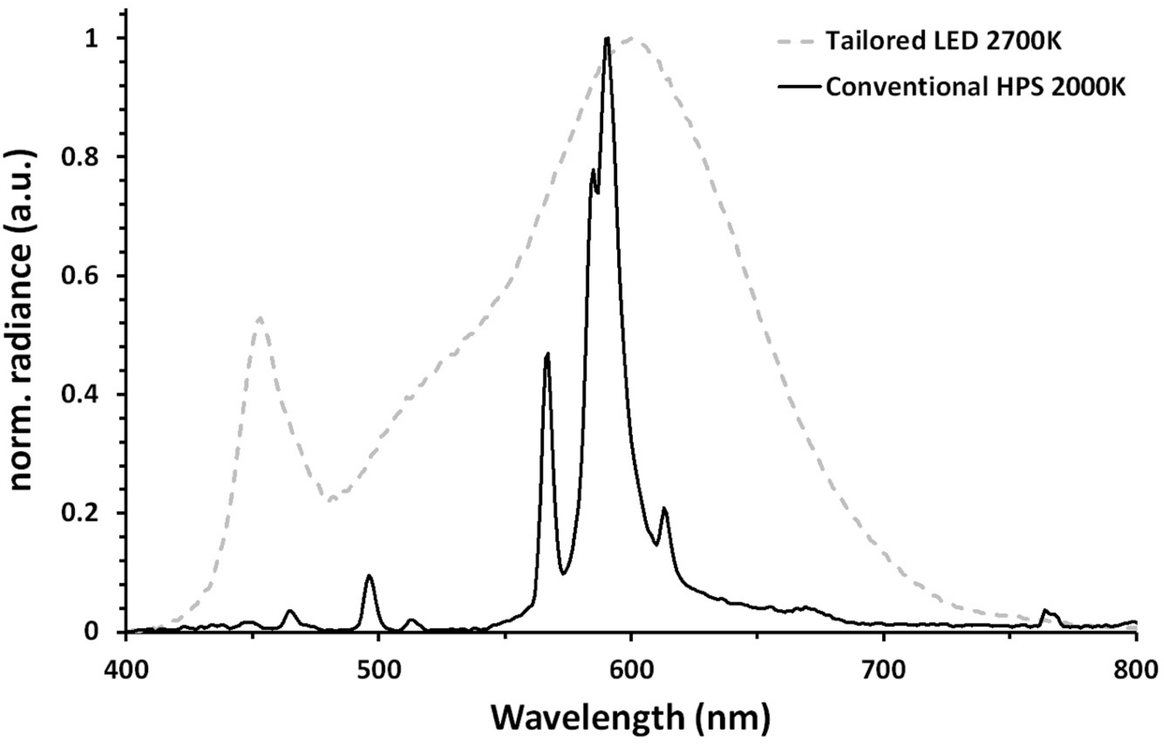


**Supplementary Figure 16: Spectra of luminaires at the Municipal Site Heimbachaue.**

Tailored and shielded luminaires (LED, $E_m$ = 5.1 lx, CCT 2700K). Conventional luminaires (HPS bow-shaped, $E_m$ = 5.1 lx, 2000K).

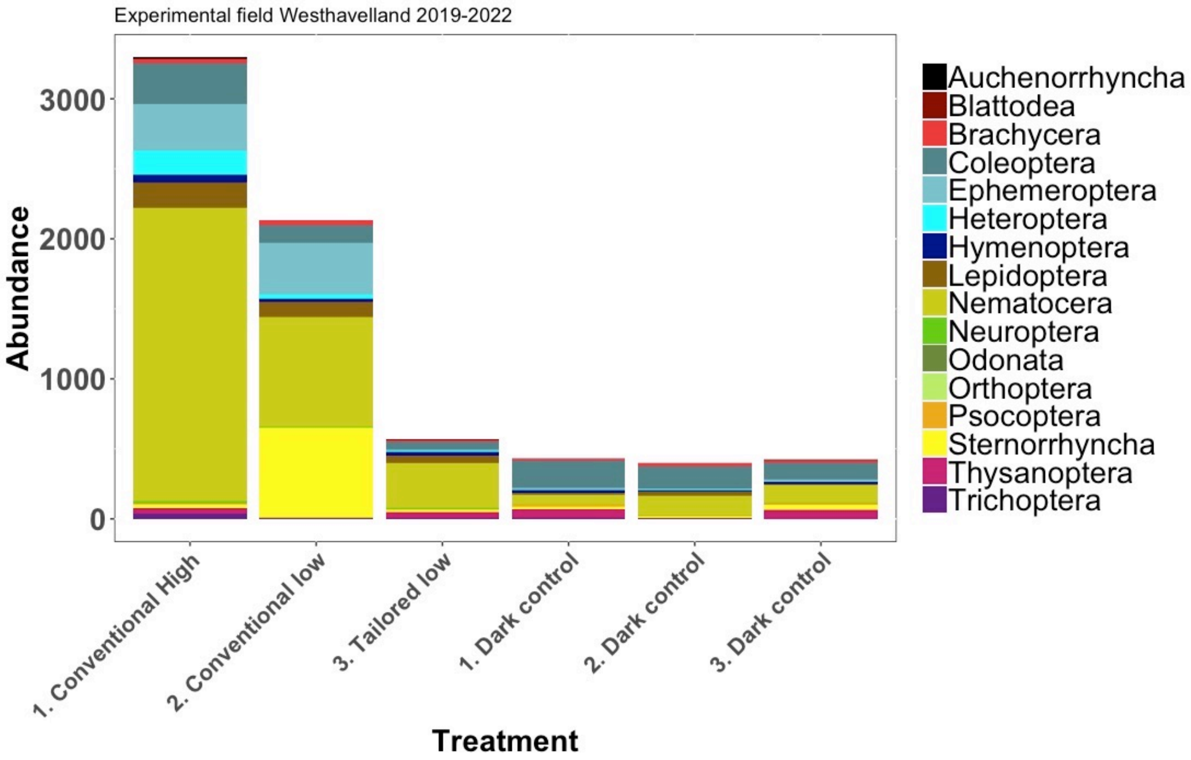


**Supplementary Figure 17: Total insect abundance (individuals) in different treatments at the experimental field site in Westhavelland.** Treatment numbers refer to treatments of the same years. 1: Conventional High 2019-2020 (n=14 samplings, LED 30.6 lx 3000K). 2: Conventional Low 2021 (n=15 samplings, LED 5.6 lx 3000K). 3: Tailored Low 2022 (n=16 samplings, LED 5.2 lx 3000K). Dark controls were sampled simultaneously to the lit treatments.

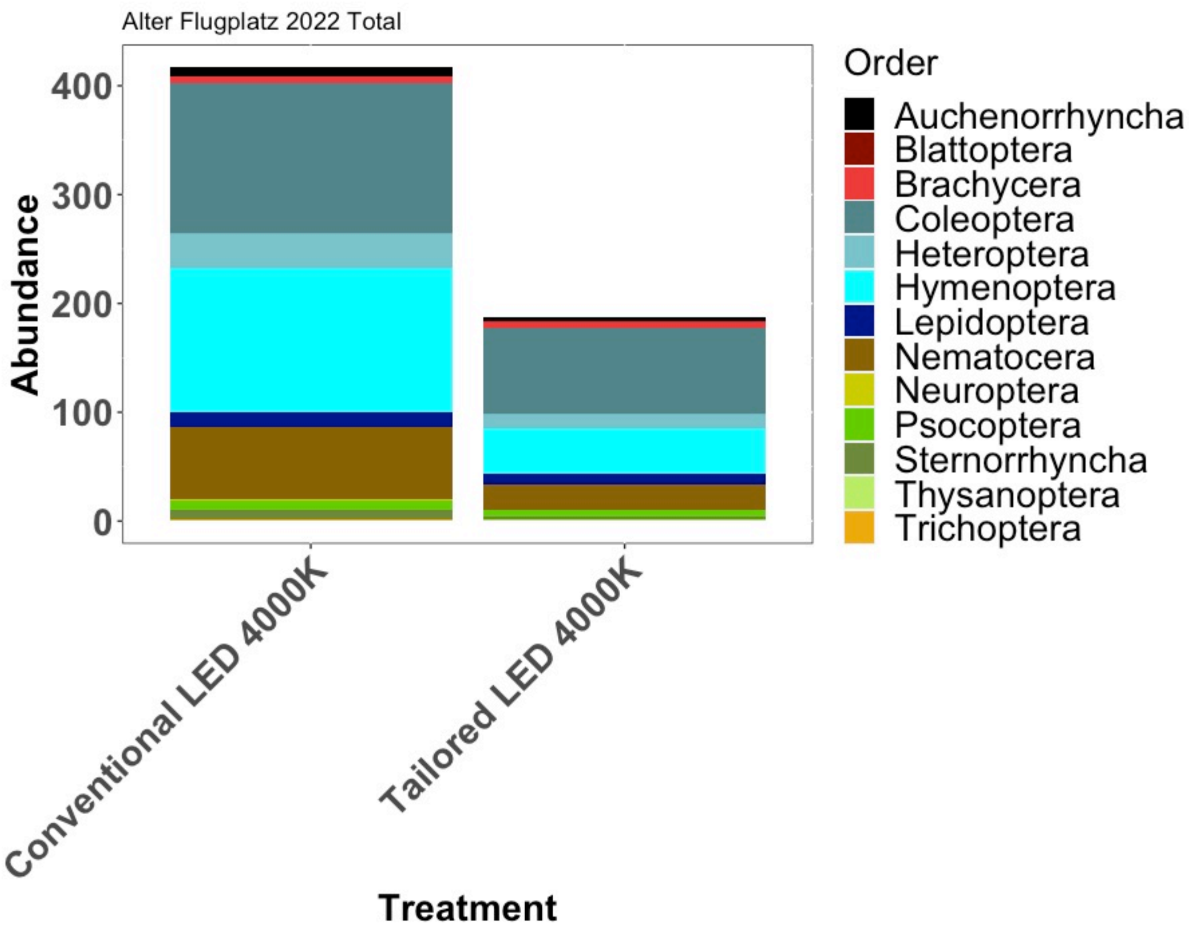


**Supplementary Figure 18: Total insect abundance (individuals) in different light treatments at the municipal site Alter Flugplatz.** n=9 samplings, 5 luminaires per treatment, transition from conventional (LED 4000K) luminaires to tailored and shielded luminaires (LED 4000K).

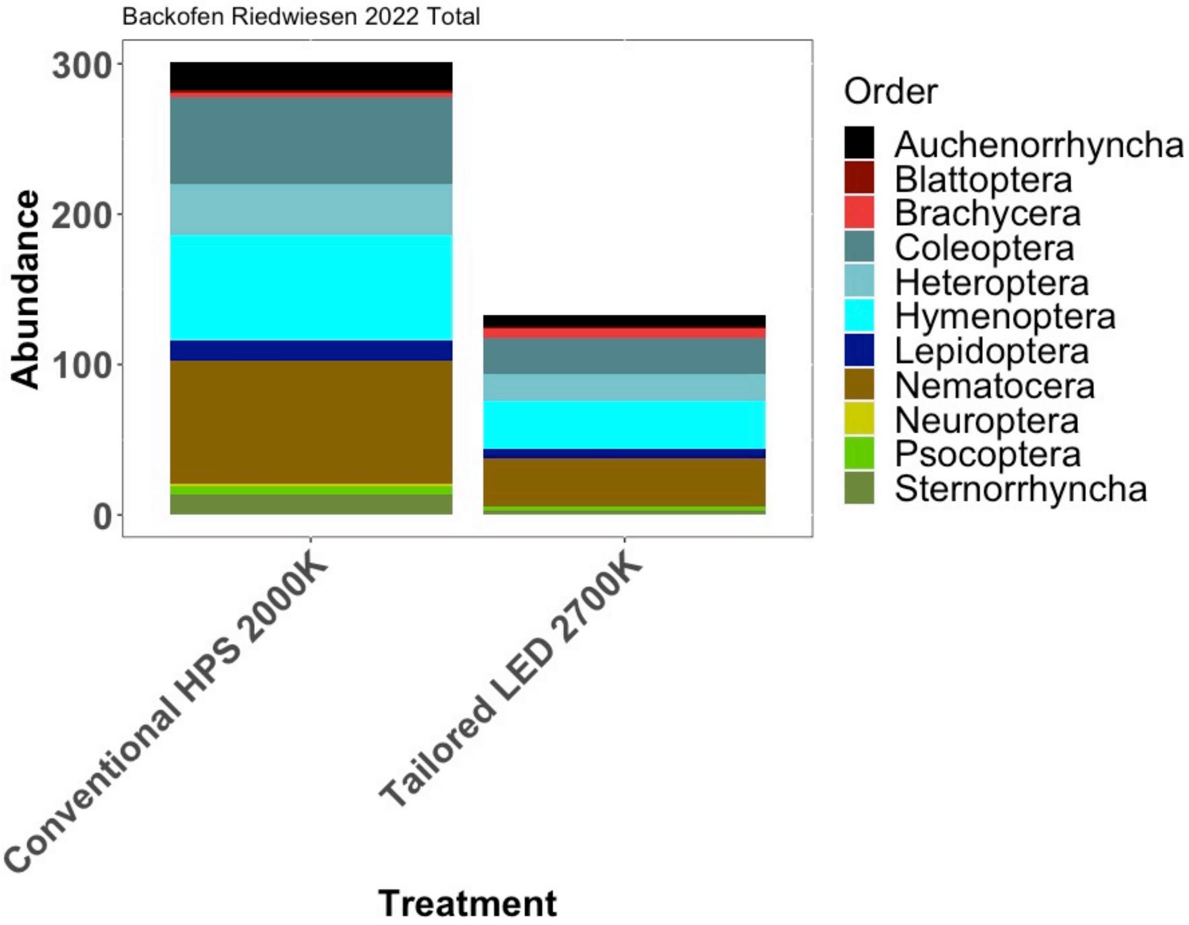


**Supplementary Figure 19: Total insect abundance (individuals) in different light treatments at the municipal site Backofen Riedwiesen.** n=7 samplings, 5 luminaires per treatment, transition from conventional (HPS 2000K cylinder) luminaires to tailored and shielded luminaires (LED 2700K).

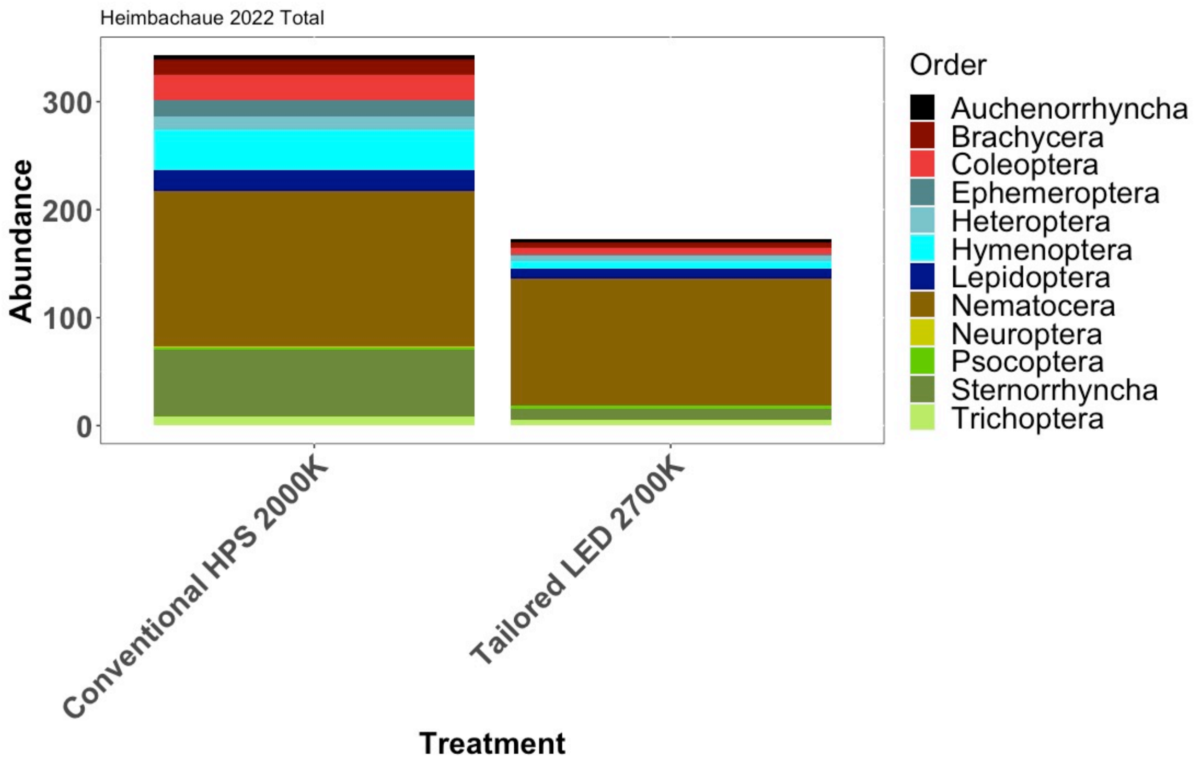


**Supplementary Figure 20: Total insect abundance (individuals) in different light treatments at the municipal site Heimbachaue.** n=7 samplings, 4 luminaires per treatment, transition from conventional (HPS 2000K bow-shaped) luminaires to tailored and shielded luminaires (LED 2700K).

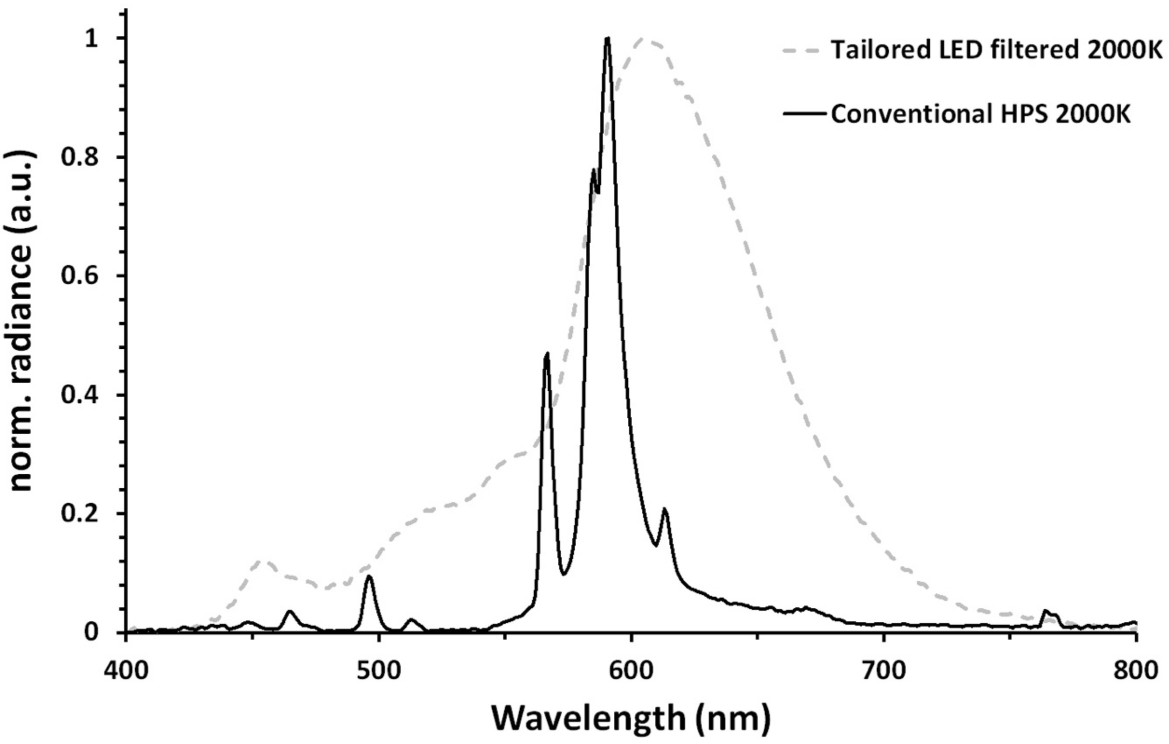


**Supplementary Figure 21: Spectra of luminaires at the Municipal Site Heimbachaue in 5 additional control nights** with conventional and tailored LED at the same CCT (2000K).

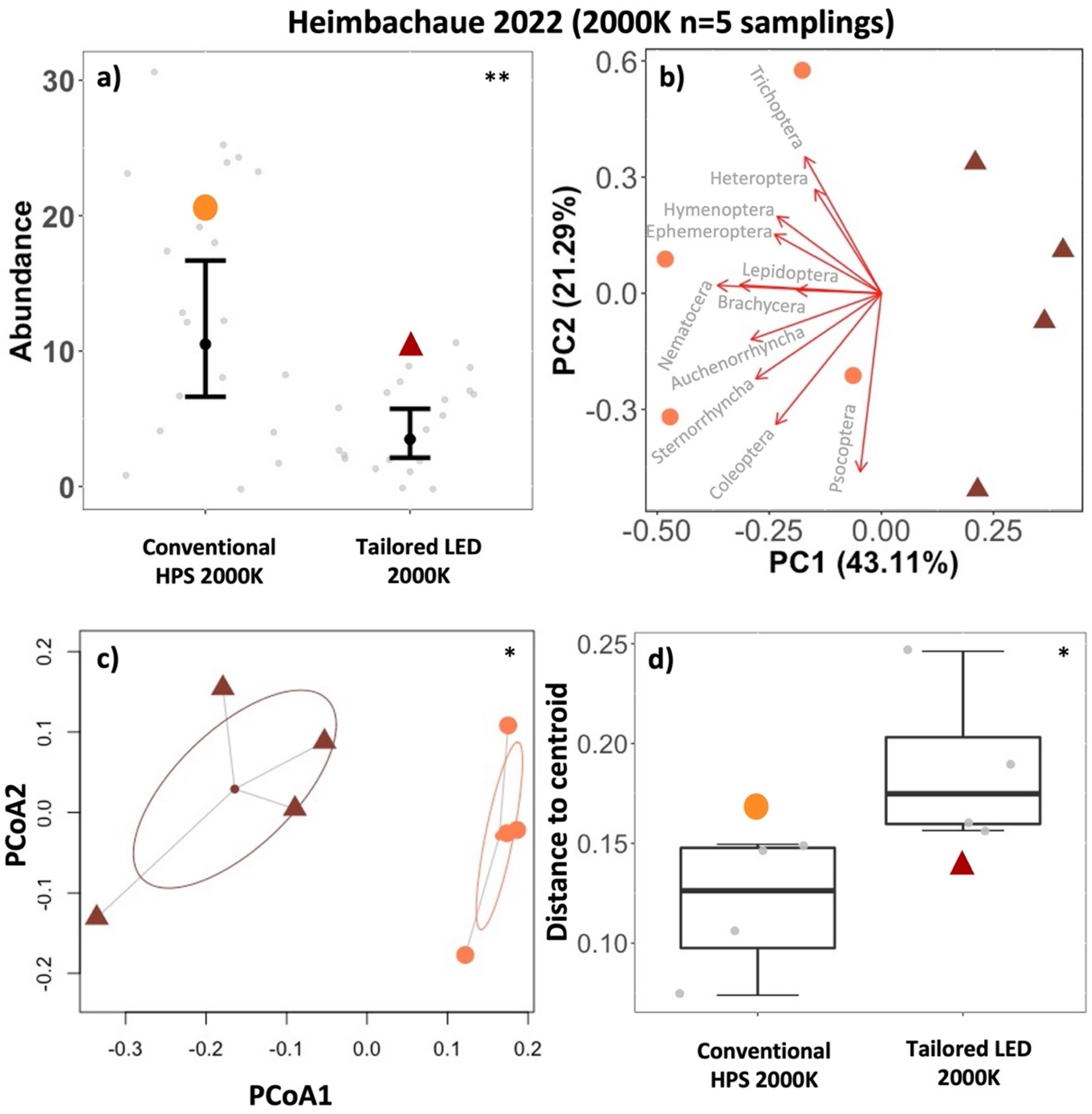


**Supplementary Figure 22: Rural municipal site, Heimbachaue, Betzweiler.** Control experiments with tailored and shielded LED with a CCT of 2000K instead of 2700K (Conventional HPS 2000K bow-shaped vs. Tailored LED 2000K) **a)** Total number of individuals per road light and night (Abundance) (grey points) in different treatments (symbols). n=5 samplings, 4 luminaires per treatment, transition from conventional luminaires (HPS 2000K) to tailored and shielded luminaires (LED 2000K). Predictions (black points) and 95% confidence intervals (error bars) based on a generalized linear regression model (p<0.001`***) (abundance ~ Luminaire + mean temperature + mean wind speed +

(1|trap_number) + (1|starting_date), family=nbinom1) **b)** PCA Principal component analysis of multiple luminaires (n=4) grouped by treatment (symbols). Arrows depict loadings of individual insect taxa **c)** PERMANOVA Permutational Multivariate Analysis of Variances (df=1, $R^2$= 0.323, F= 6.590, p= 0.035 *) of multiple luminaires grouped by treatment (symbols). Ellipses based on SD **d)** Within group dispersion (community dispersion) of luminaires in the same treatment (ANOVA p=0.045 *). Distances to centroids (grey points). Boxplots show median (thick line), interquartile range (IQR) (box), outliers (black points), 1.5 x IQR (whiskers).

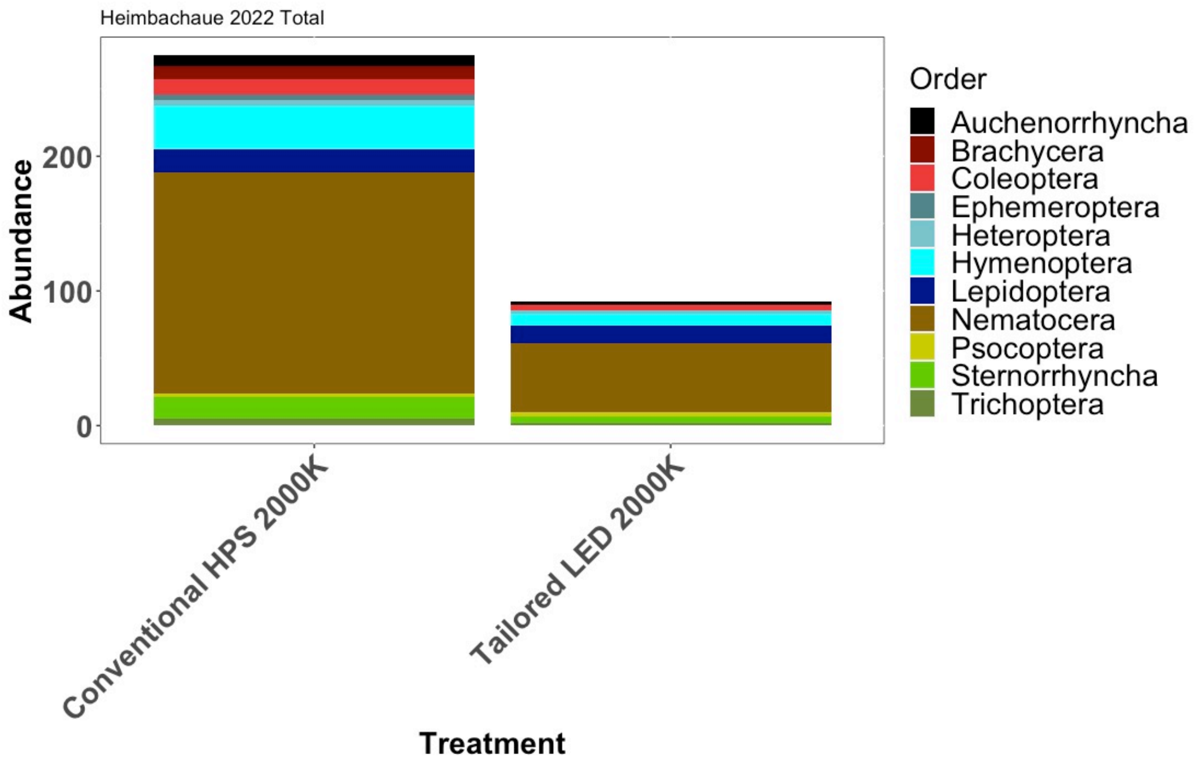


**Supplementary Figure 23: Total insect abundances in additional control experiments at the municipal site Heimbachaue with the same colour temperature between treatments (Conventional HPS 2000K bow-shaped vs. Tailored LED 2000K).** n=5 samplings, 4 luminaires per treatment, transition from conventional (HPS 2000K bow-shaped) luminaires to tailored and shielded luminaires (LED 2000K).

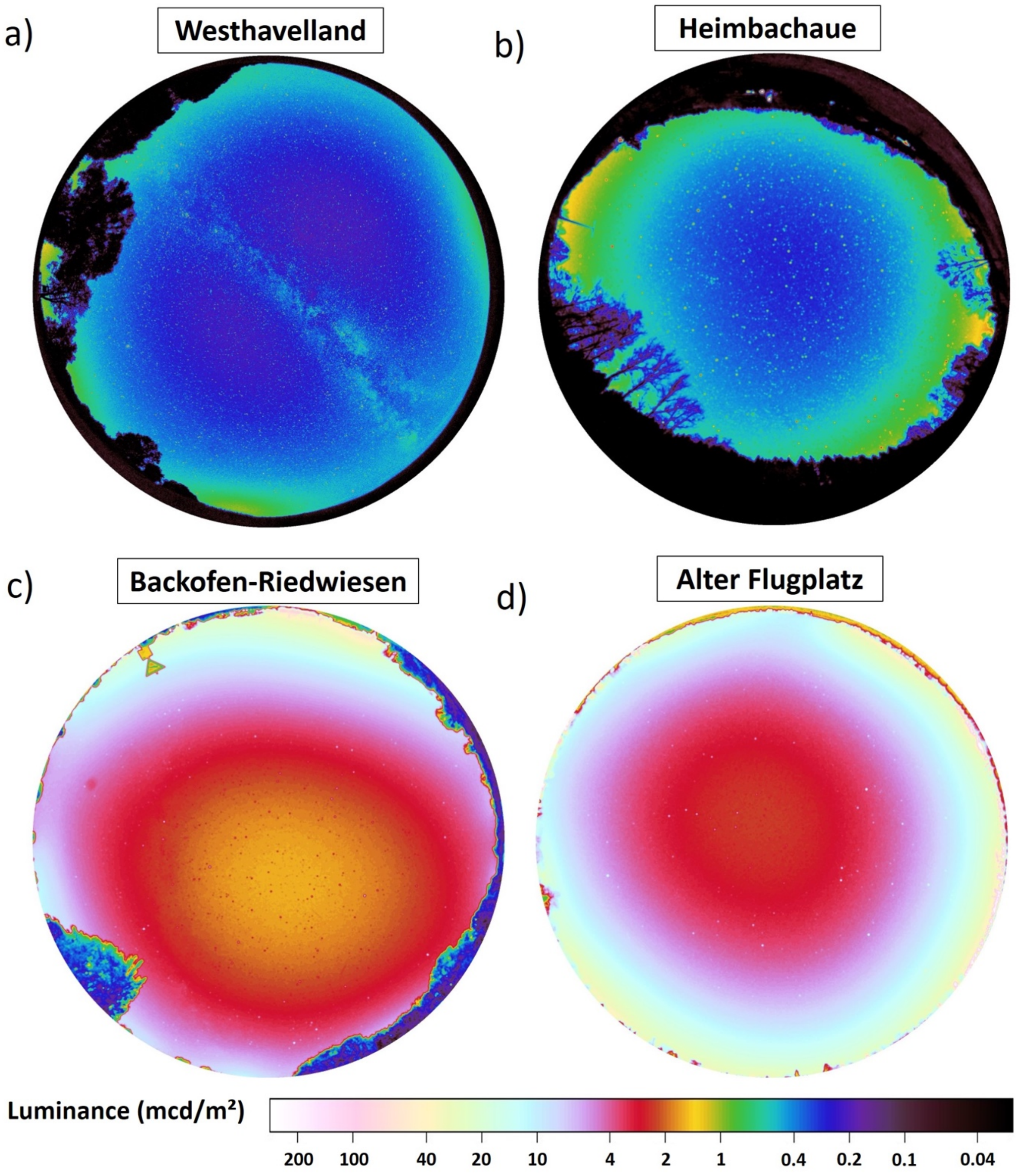


**Supplementary Figure 24: The four images show luminance maps obtained with an all-sky camera and processed with Sky Quality Camera software** (see methods). Values were categorized in terms of zenith luminance. **a)** Westhavelland with very low skyglow 0.23 mcd/m² (Milky Way subtracted), **b)** Heimbachaue with low skyglow 0.29 mcd/m², **c)** Backofen-Riedwiesen with very high skyglow 1.6 mcd/m² and **d)** Alter Flugplatz Karlsruhe with extreme skyglow 2.4 mcd/m². Photos © Andreas Jechow.

**Supplementary References**

1. Holzhauer. S. I. J. *et al.* Out of the dark: Establishing a large-scale field experiment to assess the effects of artificial light at night on species and food webs. *Sustain.* **7**. 15593–15616 (2015).
